# *GRID1*/GluD1 homozygous variants linked to intellectual disability and spastic paraplegia impair mGlu1/5 receptor signaling and excitatory synapses

**DOI:** 10.1038/s41380-024-02469-w

**Published:** 2024-02-28

**Authors:** Dévina C. Ung, Nicolas Pietrancosta, Elena Baz Badillo, Brigitt Raux, Daniel Tapken, Andjela Zlatanovic, Adrien Doridant, Ben Pode-Shakked, Annick Raas-Rothschild, Orly Elpeleg, Bassam Abu-Libdeh, Nasrin Hamed, Marie-Amélie Papon, Sylviane Marouillat, Rose-Anne Thépault, Giovanni Stevanin, Jonathan Elegheert, Mathieu Letellier, Michael Hollmann, Bertrand Lambolez, Ludovic Tricoire, Annick Toutain, Régine Hepp, Frédéric Laumonnier

**Affiliations:** 1 UMR 1253, iBrain, Université de Tours, Inserm, 37032 Tours, France; 2 Sorbonne Université, INSERM, CNRS, Neuroscience Paris Seine - Institut de Biologie Paris Seine, 75005 Paris, France; 3 Laboratoire des biomolécules, Département de chimie, École normale supérieure, PSL University, Sorbonne Université, CNRS, 75005 Paris, France; 4 Univ. Bordeaux, CNRS, IINS, UMR 5297, F-33000 Bordeaux, France; 5 Department of Biochemistry I - Receptor Biochemistry, Faculty of Chemistry and Biochemistry, Ruhr University Bochum, D-44780 Bochum, Germany; 6 The Institute for Rare Diseases, Edmond and Lily Safra Children’s Hospital, Sheba Medical Center, Tel-Hahsomer, 5262000 Israel; 7 Talpiot Medical Leadership Program, Sheba Medical Center, Tel-Hashomer, 5262000 Israel; 8 Faculty of Medicine, Tel-Aviv University, Tel-Aviv, 69978 Israel; 9 Department of Genetics, Hadassah Medical Center, Jerusalem, Israel; 10 Faculty of Medicine, Hebrew University of Jerusalem, Jerusalem, Israel; 11 Department of Pediatrics, Makassed Hospital and Faculty of Medicine, Al-Quds University, East Jerusalem, Jerusalem, Palestine; 12 Pediatric Neurology Unit, Edmond and Lily Safra Children’s Hospital, Sheba Medical Center, Tel-Hahsomer, 5262000 Israel; 13 Univ. Bordeaux, INCIA, UMR 5287 CNRS EPHE, F-33000 Bordeaux, France; 14 Unité fonctionnelle de Génétique Médicale, Centre Hospitalier Universitaire, 37044 Tours, France; 15 Service de Génétique, Centre Hospitalier Universitaire, 37044 Tours, France

**Keywords:** Neuroscience, Genetics

## Abstract

The ionotropic glutamate delta receptor GluD1, encoded by the *GRID1* gene, is involved in synapse formation, function, and plasticity. GluD1 does not bind glutamate, but instead cerebellin and D-serine, which allow the formation of trans-synaptic bridges, and trigger transmembrane signaling. Despite wide expression in the nervous system, pathogenic *GRID1* variants have not been characterized in humans so far. We report homozygous missense *GRID1* variants in five individuals from two unrelated consanguineous families presenting with intellectual disability and spastic paraplegia, without (p.Thr752Met) or with (p.Arg161His) diagnosis of glaucoma, a threefold phenotypic association whose genetic bases had not been elucidated previously. Molecular modeling and electrophysiological recordings indicated that Arg161His and Thr752Met mutations alter the hinge between GluD1 cerebellin and D-serine binding domains and the function of this latter domain, respectively. Expression, trafficking, physical interaction with metabotropic glutamate receptor mGlu1, and cerebellin binding of GluD1 mutants were not conspicuously altered. Conversely, upon expression in neurons of dissociated or organotypic slice cultures, we found that both GluD1 mutants hampered metabotropic glutamate receptor mGlu1/5 signaling via Ca^2+^ and the ERK pathway and impaired dendrite morphology and excitatory synapse density. These results show that the clinical phenotypes are distinct entities segregating in the families as an autosomal recessive trait, and caused by pathophysiological effects of GluD1 mutants involving metabotropic glutamate receptor signaling and neuronal connectivity. Our findings unravel the importance of GluD1 receptor signaling in sensory, cognitive and motor functions of the human nervous system.

## Introduction

Intellectual disability (ID) and spastic paraplegia (SPG) are central nervous system disorders with marked clinical and genetic heterogeneity [1, 2]. The association of SPG with ID or MR (mental retardation, the out of use designation of ID) is frequent with 106 and 127 entries in the OMIM (Online Mendelian Inheritance in Man) database, respectively. Conversely, the triple combination of ID, SPG and glaucoma appears only once (OMIM#278050) with description of four patients of both sexes in two sibships of a large inbred pedigree [3], and of three male siblings born to first-cousin parents [4]. Although the consanguinity and presence of affected females suggest an autosomal recessive inheritance, the genetic basis of this distinct entity is unknown.

Glutamate delta receptors GluD1 (encoded by the *GRID1* gene) and GluD2 (*GRID2* gene) belong to the family of ionotropic glutamate receptors (iGluRs), which consist in homo- or heterotetrameric arrangements of subunits, and play key roles in synaptic transmission and plasticity [5–7]. GluDs do not bind glutamate but, instead, the binding of cerebellin and D-serine on distinct extracellular domains cooperatively gate GluD ion channels, whose opening is alternatively triggered by activation of Gq-coupled metabotropic glutamate receptors (mGlu1/5), or α1-adrenergic receptors [8–11]. The binding of these ligands also triggers or modulates metabotropic signals, cerebellin additionally enabling postsynaptic GluDs to participate in excitatory synapse formation/stabilization via attachment with presynaptic neurexin [6, 7, 12–14]. GluD1 and GluD2 are widely expressed in the brain at excitatory postsynaptic sites, GluD1 predominating over GluD2 outside the cerebellum [15–17]. The implication of *GRID1* in pathology is suggested by association of *GRID1* variants with risk of neuropsychiatric disorders [18–26], and by alterations observed in *Grid1*^*−/−*^ mice at behavioral, cognitive, synaptic, and mGlu1/5 signaling levels [13, 27–31]. Yet, truly pathogenic *GRID1* variants have not been described in human disease so far.

Here, we report the identification of homozygous missense variants in the *GRID1* gene by genome-wide linkage analysis and/or whole exome sequencing (WES) in siblings from two unrelated consanguineous families presenting with mild or moderate ID, non- or slowly-progressive SPG, with (p.Arg161His) or without (p.Thr752Met) diagnosis of open angle glaucoma. Molecular modeling and experimental studies indicated that the mutations alter structural interactions within GluD1 extracellular domains, impact the D-serine binding site, and impair GluD1 effects on mGlu1/5 signaling, dendrite morphology, and excitatory synapse density in rodent neurons.

## Materials and methods

Detailed materials and methods are provided as Supplementary Information

### Patients

Written informed consent for genetic analysis was obtained from all participants or their legal guardians according to the Declaration of Helsinki and following Institutional Review Board (IRB)-approved protocols in the Centre Hospitalier Universitaire de Tours (Family A) and the Hadassah Medical Center (Family B).

### Animals

Animal breeding and euthanasia were performed in accordance to European Communities Council Directive 86/609/062. *Grid1* KO mice [32] (gift from Jian Zuo, Memphis, TE, USA) and *Grid1*^−/−^ embryos obtained from heterozygous parents were genotyped as described [16]. Wild-type (WT) mice were purchased from Janvier Labs. All mice had C57BL/6 background.

### Genome wide-linkage analysis and whole exome sequencing

Genotyping of Family A was performed on Genechip® human 250 K NspI array (Affymetrix) according to manufacturer’s instructions. Whole exome sequencing (WES) was performed using SureSelect Human All Exon kit (Agilent technologies) and the HiSEQ 2000 sequencer (Illumina). For Family B, DNA sample of the proband was shipped to Otogenetics, USA (CLIA lab). Sequencing data were aligned with the Human reference genome (hg19). Genetic segregation of the candidate variant with the disease was confirmed by Sanger sequencing.

### Molecular modeling of GluD1 mutants structure

The protein was generated using Rat GluD1 receptor in complex with 7-chloro-kynurenate and Ca^2+^ as structure templates [33] (PDB codes: 6KSS and 6KSP), and prepared in the CHARMM-GUI web server [34] to insert protein in a membrane and solvate with water and ions. Mutant models were generated using Built Mutant protocol from Discovery Studio 2019. Molecular docking experiments of D-Serine, glycine and kynurenic acid at the active site were performed as described [35, 36]. Molecular dynamics were performed using NAMD protocols.

### Plasmids and viruses

Plasmids encoding mouse WT GluD1 (GluD1^WT^) and GluD1 variants, rat mGlu1a, Green Fluorescent Protein (GFP), or tdTomato were used for transfection of HEK293 cells or neurons. Some constructs comprised a hemagglutinin (HA) epitope inserted after the predicted signal peptides of GluD1 and mGlu1a, this latter additionally comprising the Venus GFP variant fused to its C-terminus [9, 37]. Recombinant lentiviruses co-expressing GluD1 isoforms together with GFP were generated as described [9] and pseudo-virions produced at the Necker Institute Viral Vector and Gene Transfer facility (IFR94, Paris, France). Recombinant sindbis virus encoding the Ca^2+^ sensor Twitch-2B [38] was produced as described [39].

### HEK293T cells

HEK293T cells (ATCC Number: CRL-3216) culture, plasmid transfection, membrane protein extraction, immunoprecipitation, western blotting and immunocytochemistry were performed using standard techniques and antibodies listed in Supplementary Table. For cerebellin binding experiments, HEK cells expressing GluD1 or GluD1 variants were incubated with 20 µg/ml recombinant human HA-tagged Cerebellin 1 (Cbln1, Biotechne 6934-CB-025) prior to rinsing and immunostaining.

### Primary cortical or hippocampal cell cultures

Primary cortical or hippocampal cell cultures were prepared and cultured as described [40] from E17-E18 *Grid1*^−/−^ or *Grid1*^+/+^ mice embryos, respectively. Western blotting and immunocytochemistry were performed using standard techniques and antibodies listed in Supplementary Table. For analyses of mGlu1/5 signaling, cortical cell cultures were transduced at 10 days in vitro (DIV) with GluD1-expressing lentiviruses, cultured for 4–7 additional days. Cultures were then either tested for ERK activity or transduced with Twitch-2B expressing sindbis virus the day before Ca^2+^ imaging experiments. ERK activity was measured on cultures incubated for 1 hour in medium containing 300 nM TTX (Latoxan) and 50 µM of the NMDAR antagonist APV (Hello Bio), then incubated in the presence or absence of RS-3,5-dihydroxyphenylglycine 2 (DHPG, 100 µM, Hello Bio), and finally processed for western blotting. Ca^2+^ imaging was performed in a perfusion chamber and responses to S-DHPG (50 µM, Hello Bio) monitored using a custom-built 2-photon laser scanning microscope as described [41]. For analyses of neurites and excitatory synapses, primary hippocampal cell cultures were transfected at DIV4 (dendritic morphometry) or DIV11-DIV13 (spines and synapses). Cultures were next incubated for 48 h, and then processed for immunocytochemistry.

### Statistical analyses

All experiments were repeated at least three times. When d’Agostino-Pearson normality tests were successfully passed, we used One-way ANOVA parametric test, followed by Tukey’s post hoc method. For samples that did not pass the normality test, we used Kruskal–Wallis method followed by Dunn’s post hoc test. Results are given as mean ± standard error of the mean. Differences were considered significant if *p* < 0.05.

## Results

### Clinical description of the families

**Fig. 1:**
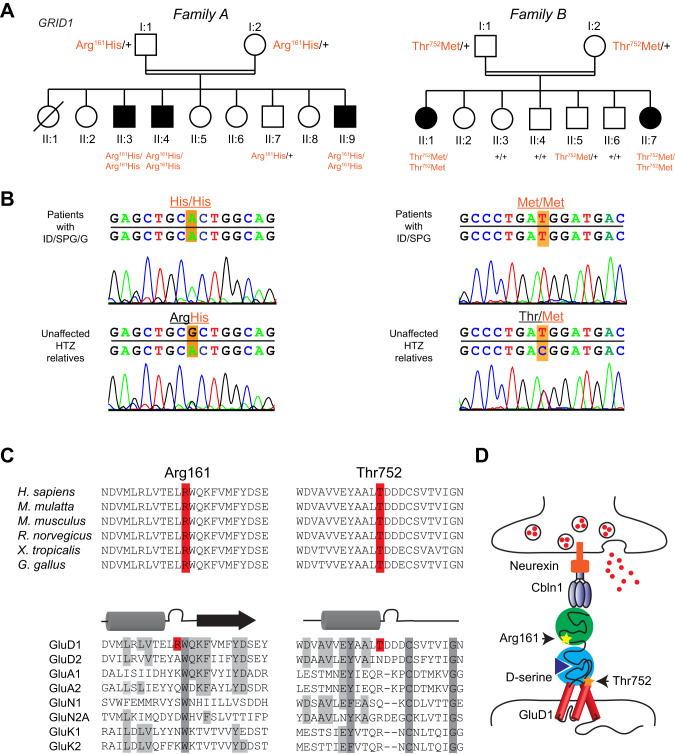
Homozygous *GRID1* variants p.Arg161His and p.Thr752Met causing ID and SPG with or without Glaucoma. **A** Pedigree of the families. Filled symbols indicate individuals with ID, SPG and Glaucoma (***left***), or ID and SPG (***right***). Individual number is indicated below each symbol. **B** Sanger sequencing electrophoregrams showing the *GRID1* homozygous missense mutations c.482G>A, p.Arg161His and c.2255C>T, p.Thr752Met in the affected patients and the heterozygous mutations in unaffected relatives. **C** Amino-acid alignments showing conservation of GluD1 R^161^ and T^752^ residues across species, but not among iGluR family members. **D** Spatial organization of the transsynaptic complex GluD1-cerebellin (Cbln1)-neurexin at the glutamatergic synapse. Note that R^161^ and T^752^ residues belong to cerebellin-binding (ATD) and D-serine-binding domains (LBD), respectively.

Family A included three affected brothers born to first-degree cousins (Fig. 1A), and presenting with non- or slowly-progressive SPG diagnosed in infancy with no other neurological signs, mild/moderate ID with normal occipitofrontal circumference, and juvenile open angle glaucoma causing severe visual impairment. This clinical picture is strikingly similar to earlier descriptions of this syndrome [3, 4]. Brain MRI, electromyography, metabolic investigations in one patient, and standard chromosome analysis of the three brothers were normal. Linkage to genes *ARX*, *XNP*, *PLP* and *L1CAM* was excluded, sequencing of *MECP2* did not detect a causative variant, and high-resolution array-Comparative Genomic Hybridization (CGH) analysis did not reveal pathogenic copy number variation related to the disease.

Family B included two affected sisters, born to consanguineous parents (Fig. 1A). The proband presented at 6 years of age with global developmental delay, spastic paraplegia, craniosynostosis, dysmorphic features (brachycephaly, bilateral ptosis), and minor skeletal anomalies. Her 24-year old sister was similarly affected, but also showed kyphosis of the cervical spine, and required a wheelchair. Ophthalmologic examination could not be performed on either sister. The clinical features for Family B are detailed in Supplementary Information. Initial genetic investigations for the proband of Family B included chromosomal karyotype analysis which was normal, as well as CGH analysis, which was considered normal but notable for an intronic 50Kb deletion in 7q36.2 encompassing the *DPP6* gene (arr:7q36.2(153,921,762-153,951,944)X1).

The clinical features for the five affected individuals are summarized in Table 1, together with side-by-side comparison with earlier observations [3, 4].

**Table 1:** Clinical and genetic features of patients with ID and SPG with or without glaucoma, from present and earlier reports.

	From Heijbel and Jagell [3]; Chenevix-Trench et al. [4]	Family A			Family B	
*Individuals*	7 patients	1	2	3	4	5
Gender	5 M - 2 F	M	M	M	F	F
Ethnicity	4 Swedish - 3 French Canadian	Algerian	Algerian	Algerian	Arab-Muslim	Arab-Muslim
Parental consanguinity	Y	Y	Y	Y	Y	Y
Age at evaluation (years)	Adults (to 73)	24	40	37	6	24

*Neurological features*						
Developmental delay / ID	ID (3 mild, 3 moderate, 1 severe)	ID (mild)	ID (mild IQ = 50)	ID (moderate IQ = 40)	GDD	ID
Gross motor abilities	6/7 able to walk	Walking without aid	Walking with canes	Walking without aid	Cannot run or climb stairs	Unstable walking, needs wheelchair
Age of walking acquisition	4–10	4–5	4–5	4–5	2.5	4
Spastic paraplegia −onset/diagnosis −progression	Y (7/7) 1^st^ year of life N (3/7), very slow (4/7)	Y Birth N	Y 1^st^ year N	Y 1^st^ year N	Y NA NA	Y NA NA
Brain magnetic resonance imaging findings	NA	Normal	NA	NA	Mild diffuse cortical atrophy	NA

*Ophthalmological involvement*						
Glaucoma −Age at diagnosis −Surgery/complications	Y (7/7) 14–34 NA	Y 24 LE optic atrophy	Y 20 Optic atrophy	Y NA Y	NA	NA
Vision	7/7 severe impairment	LE poor vision	RE poor vision LE blindness	Blindness	NA	NA

*Other observations*						
Dysmorphic features	N	N	N	N	Y^a^	Y^a^
Skeletal involvement	N	N	N	N	Y^a^	Y^a^
Additional features	N	N	N	N	Sparse reddish hair	

*GRID1 variant information*						
Genomic (hg19)	NA	chr10:87966159C>T			chr10:87379729G>A	
cDNA (NM_017551.2)	NA	c.482G>A			c.2255C>T	
Protein	NA	p.(Arg161His)			p.(Thr752Met)	
Inheritance	NA	Homozygous (parents unaffected)			Homozygous (parents unaffected)	
Sequencing method	NA	WES		Sanger	WES	Sanger

*GDD* global developmental delay, *ID* intellectual disability, *IQ* intellectual quotient, *LE* left eye, *N* no, *NA* not available, *RE* right eye, *WES* whole exome sequencing, *Y* yes.

^a^Supplementary Information.

### Identification of homozygous variants in *GRID1*

As consanguinity in both family pedigrees suggests autosomal recessive inheritance (Fig. 1A), we performed genome-wide single nucleotide polymorphism (SNP) genotyping for Family A in the three affected brothers, one healthy brother and both parents. Two homozygous regions with significant linkage were found at chromosome 10q23.1-q25.2 region (30.6 Mb between rs11201697 and rs7077757 markers, see Supplementary Fig. S1) and at chromosome 12q24.33 region (161 kb between rs10773690 and rs4759984 markers). WES analysis on two affected brothers and their father allowed the identification of a homozygous missense mutation of the *GRID1* gene (NM_017551.2: c.482G>A, p.Arg161His; hg19, chr10:87966159C>T) within the 10q22q23 candidate region. This variant segregated in an autosomal recessive manner in all affected members of Family A (Fig. 1A, B, Table 1). The p.Arg161His *GRID1* variant, predicted as “Disease causing” by *Mutation Taster* (score 0.9697) and referred in dbSNP (rs771100097), is only found at heterozygous state in 4 individuals from gnomAD database (Minor Allele Frequency, MAF = 1.60e−5).

For Family B, single (proband-only) WES was pursued, and brought to the identification of a homozygous missense variant in *GRID1* (NM_017551.3: c.2255C>T, p.Thr752Met; hg19, chr10:87379729G>A). Using Sanger sequencing, this variant was confirmed to segregate with the disease in Family B, with both affected sisters homozygous for the variant, both parents and an unaffected male sibling found to be heterozygous carriers, and three additional unaffected siblings wild type for the variant (Fig. 1A, B, Table 1). The p.Thr752Met variant is only found at heterozygous state, in 11 individuals from gnomAD database (MAF = 3.89e−5).

Finally, sequencing of a cohort of more than 200 patients affected with SPG, isolated or associated with ID, failed to identify additional variants in *GRID1*.

### Impact of Arg161His and Thr752Met mutations on GluD1 extracellular domains

**Fig. 2:**
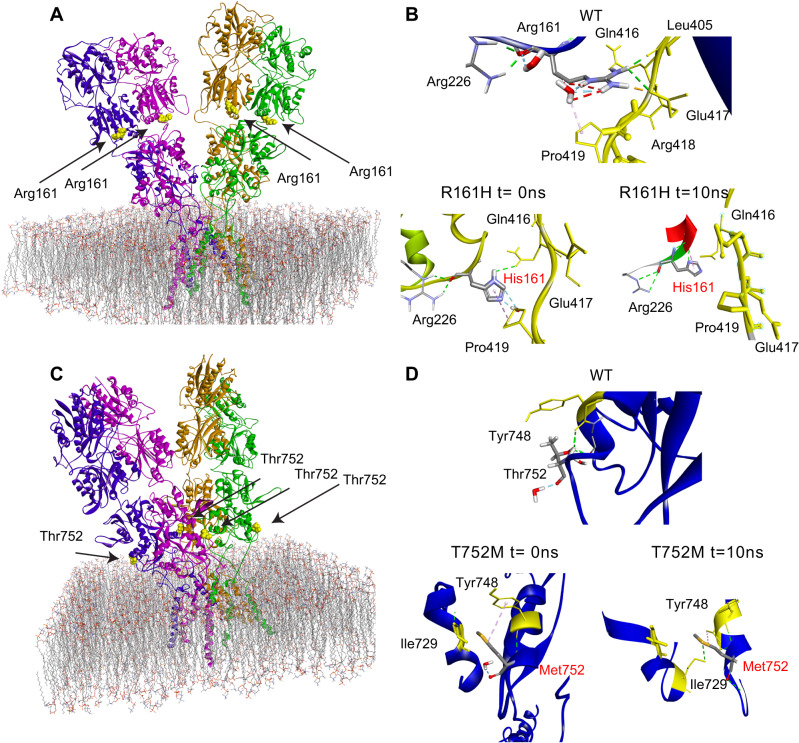
Modeling the structural impact of GluD1 R^161^H and T^752^M mutations on cerebellin-binding and D-serine-binding domains. **A**, **C** Structure of the GluD1 homotetramer sitting above the plasma membrane - adapted from ref. [33]. Mutations affect residues situated at the interface (R^161^) between ATD and LBD extracellular domains, or within LBD (T^752^). **B**, **D** Predicted interactions of wt R^161^ and T^752^ residues, and of mutant H^161^ and M^752^ residues. The R^161^H mutation suppresses interaction with D^417^ residue of the loop linking ATD to LBD, thereby changing loop conformation (t = 0 ns). These interactions were also decreased during molecular dynamics (t = 10 ns), highlighting the weakness of ATD-LBD interactions in mutant protein. The T^752^M mutation results in supplementary interaction with I^729^ and Y^748^ residues of the LBD, thereby rigidifying this latter domain. These additional interactions, preserved during molecular dynamics, lead to a stiffening of LBD.

The p.Arg161His (R^161^H) and p.Thr752Met (T^752^M) mutations concern GluD1 amino acid residues conserved among vertebrate species, but not among iGluR subunits (Fig. 1C), consistent with functional heterogeneity within this family [5, 33, 42]. Based on GluD1 3D structure [33], we assigned R^161^ and T^752^ residues to extracellular Amino Terminal Domain (ATD) and Ligand Binding Domain (LBD), which bind cerebellin and D-serine, respectively (Fig. 1D), the R^161^ residue being at the ATD-LBD interface distant from cerebellin binding residues, and the T^752^ residue lying within the LBD (Fig. 2A, C). We modeled the complete GluD1 structure by generating unresolved 3D loops crucial for activation [33] (Supplementary Fig. S2), and characterized effects of the mutations on this model. We found that the R^161^H mutation impacts the hinge between ATD and LBD by modifying the binding pattern with Q^416^, D^417^, and P^419^ residues of the loop linking the two domains, with possible consequences on their cooperativity [11, 33, 43]. In molecular dynamics experiments, the interaction patterns of H^161^ with Q^416^, E^417^ and P^419^ were not fully conserved over time (Fig. 2B), arguing for instability of ATD-LBD interaction in the mutant protein. The T^752^M mutation results in additional interactions between M^752^, Y^748^ (in the same α helix) and I^729^ (in adjacent α helix) that could lead to a stiffening of LBD. Further modeling experiments on the mutant protein showed a conservation of these interactions (i.e., M^752^ with Y^748^ and I^729^) over molecular dynamics duration and support a stiffening of the LBD. (Fig. 2D). Molecular docking indicated that D-serine affinity is decreased in GluD1^T752M^, but little modified in GluD1^R161H^ (binding energy: wild-type GluD1 (GluD1^WT^), −107.9; GluD1^R161H^, −102.7; GluD1^T752M^, −68.8 kJ/mole), whereas binding of endogenous ligand glycine and synthetic ligand 7-chloro-kynurenate [44] are weakened by both mutations (from −100.7 and −108.8 kJ/mole in GluD1^WT^, to −76.8 and −79.1 kJ/mole in GluD1^R161H^, and to −65,7 and −77.8 kJ/mole in GluD1^T752M^, respectively). Electrophysiological analyses of the ion current mediated by a constitutively open channel GluD1 isoform [45] revealed that modulation of this current by D-serine and glycine was impaired by the T^752^M mutation, but moderately affected by the R^161^H mutation (Supplementary information and Supplementary Fig. S3), consistent with predictions of molecular modeling. These results indicate that R^161^H and T^752^M mutations can both affect GluD1 function by altering ligand binding and/or its transduction to transmembrane/intracellular signaling.

### The R^161^H and T^752^M mutations do not hamper cerebellin binding to GluD1

To gain insight into the functional consequences of the mutations, we first expressed GluD1^WT^, GluD1^R161H^, and GluD1^T752M^ in HEK cells, and found that GluD1 amount, molecular weight, and membrane insertion were not conspicuously affected by the mutations (Supplementary Fig. S4A). Transfection of hippocampal primary cell cultures confirmed plasma membrane expression of all variants in putative excitatory neurons with similar distribution along dendritic shafts and spines (Supplementary Fig. S4B). Hence, the pathogenicity of GluD1^R161H^ and GluD1^T752M^ does not result from deficits in their expression, stability or trafficking. We next compared the ability of GluD1^WT^, GluD1^R161H^ and GluD1^T752M^ to bind extracellular Cerebellin-1 in HEK cells, and found that both mutants retained the cerebellin-binding capability of GluD1^WT^ (Supplementary Fig. S5). This was confirmed using Bio-Layer Interferometry measurements of interactions of recombinant Cerebellin-1 with WT or mutant GluD1 extracellular domains, which did not reveal differences in interaction kinetics or affinity that may hamper cerebellin binding to GluD1 mutants (Supplementary information and Supplementary Fig. S6). Hence, cerebellin binding, and thus trans-synaptic scaffolding ability [6, 12, 14], is essentially preserved in both mutants, consistent with R^161^H and T^752^M mutations being distant from cerebellin-binding residues in the GluD1 3D structure.

### The R^161^H and T^752^M mutations impair the modulation of mGlu1/5 signaling by GluD1

Our above results suggest that R^161^H and T^752^M mutations can affect GluD1 function by altering ligand binding and its transduction to transmembrane/intracellular signaling [6, 7, 11, 14]. We thus searched for alteration of mGlu1/5 signaling, which involves GluD1, is impaired in *Grid1*^*−/−*^ mice, and whose dysregulation at the level of non-canonical pathways tightly relates to ID and related neurodevelopmental disorders [9, 30, 46–48].

We first verified that mGlu1 co-immunoprecipitated with GluD1^WT^, GluD1^R161H^, or GluD1^T752M^ with similar efficiency upon co-expression in HEK (Supplementary Fig. S7), indicating that the mGlu1-GluD1 physical interaction [9, 30] is not impaired by the R^161^H and T^752^M mutations.

**Fig. 3:**
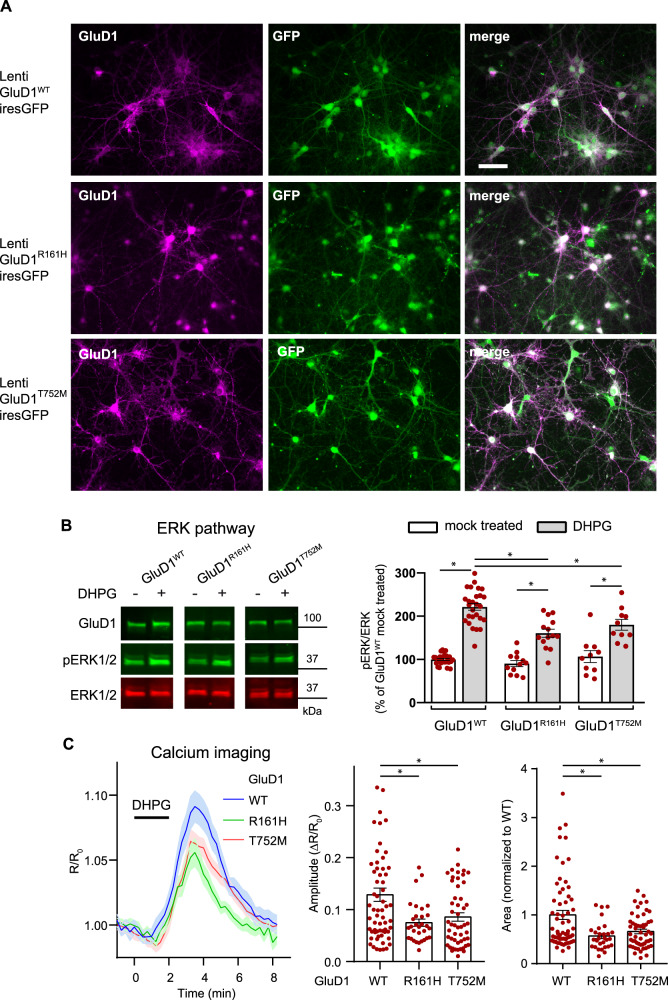
The R^161^H and T^752^M mutations hamper the modulation of mGlu1/5 signaling by GluD1. **A** Fluorescence pictures of primary cortical cell cultures from *Grid1*^−/−^ mouse co-expressing GluD1^WT^/GluD1^R161H^/GluD1^T752M^ and GFP following lentiviral transfer, scale bar: 50 µm. **B** Western blot analysis of virally transduced cortical cultures following incubation in presence or absence of the mGlu1/5 agonist DHPG (100 µM) and graph summarizing results obtained in mock-treated or DHPG-treated cultures expressing GluD1^WT^ (*n* = 27 and 27, respectively), GluD1^R161H^ (*n* = 12 and 14, respectively), or GluD1^T752M^ (*n* = 10 and 10, respectively). **C** 2-photon Ca^2+^ imaging in cortical cell cultures co-expressing GluD1^WT^/GluD1^R161H^/GluD1^T752M^ and the ratiometric Twitch-2B sensor following dual lenti/sindbis viral transfer. Traces show mean (lines) ±SEM (shade) changes of Twitch-2B YFP/CFP ratio in response to S-DHPG (50 µM), indicative of transient Ca^2+^ increase in somata of neurons expressing GluD1^WT^ (*n* = 63), GluD1^R161H^ (*n* = 31), or GluD1^T752M^ (*n* = 51). Graphs summarize results obtained from cells of at least 6 coverslips and 2 cultures per condition. *Significant differences.

Next, GluD1^WT^, GluD1^R161H^, or GluD1^T752M^ were co-expressed with GFP through lentiviral transduction in primary cultures of cortical cells from *Grid1*^−/−^ mice, which avoid influence of endogenous GluD1^WT^ on mGlu1/5 signaling. All GFP-labeled transduced cells examined were GluD1-immunopositive (Fig. 3A), and the vast majority of neurons in these cultures were transduced (Supplementary Fig. S8). In a first set of experiments, cultures were incubated 5 min in the presence/absence of the mGlu1/5 agonist DHPG (100 µM), and then processed for western blot and immunoquantification of the phosphoERK/ERK ratio (Fig. 3B). In GluD1^WT^-expressing cultures, DHPG treatment strongly increased the phosphoERK/ERK ratio relative to mock-treated control cultures (DHPG: 221 ± 8% of control; *n* = 27 control, *n* = 27 DHPG-treated cultures, Fig. 3B). The same paradigm elicited a significantly smaller increase of phosphoERK/ERK ratio in GluD1^R161H^-expressing (DHPG: 161 ± 9% of control, *n* = 14 DHPG-GluD1^R161H^) or GluD1^T752M^-expressing cultures (DHPG: 180 ± 12% of control, *n* = 10 DHPG-GluD1^T752M^; Fig. 3B). In a second set of experiments, live imaging of Ca^2+^responses of individual neurons to S-DHPG (50 µM) was performed using the ratiometric fluorescent sensor Twitch-2B additionally expressed through Sindbis viral transfer. Drug application elicited a transient increase of Twitch-2B fluorescence ratio, indicative of intracellular Ca^2+^ increase. Changes of fluorescence ratio had a significantly larger peak amplitude in GluD1^WT^- (13 ± 1%, *n* = 63), than in GluD1^R161H^- (7 ± 1%, *n* = 31) and GluD1^T752M^– expressing neurons (9 ± 1%, *n* = 51). Moreover, Twitch-2B response integral was significantly reduced in GluD1^R161H^- and GluD1^T752M^–expressing neurons (to 57 ± 5 and 66 ± 5%, respectively) compared to GluD1^WT^-expressing neurons (Fig. 3C). These results indicate that the modulation by GluD1 of mGlu1/5 intracellular signaling via both Ca^2+^ and the ERK pathway is impaired by the R^161^H and T752M mutations.

### The GluD1 R^161^H and T^752^M mutations impair dendrite morphology and excitatory synapse density

Alterations of dendrites and synapses are found in ID and related neurodevelopmental disorders in humans and mouse models [49–51]. We thus co-transfected plasmids encoding GluD1^WT^, GluD1^R161H^, or GluD1^T752M^ together with a GFP-encoding plasmid in hippocampal primary cell cultures from *Grid1*^+/+^ mice and examined the morphology of GFP-expressing neurons. The vast majority of GFP-positive neurons also over-expressed either GluD1^WT^ (96.8 ± 4.2%, *n* = 252), GluD1^R161H^ (96.3 ± 3.9%, *n* = 246), or GluD1^T752M^ (96.1 ± 4.7%, *n* = 250). Moreover, plasmid-driven expression of GluD1^WT^ and mutants was largely superior to that of endogenous GluD1 (Supplementary Fig. S9), allowing the effects of recessive R^161^H and T^752^M mutations to be evaluated in transfected *Grid1*^+/+^ neurons.

**Fig. 4:**
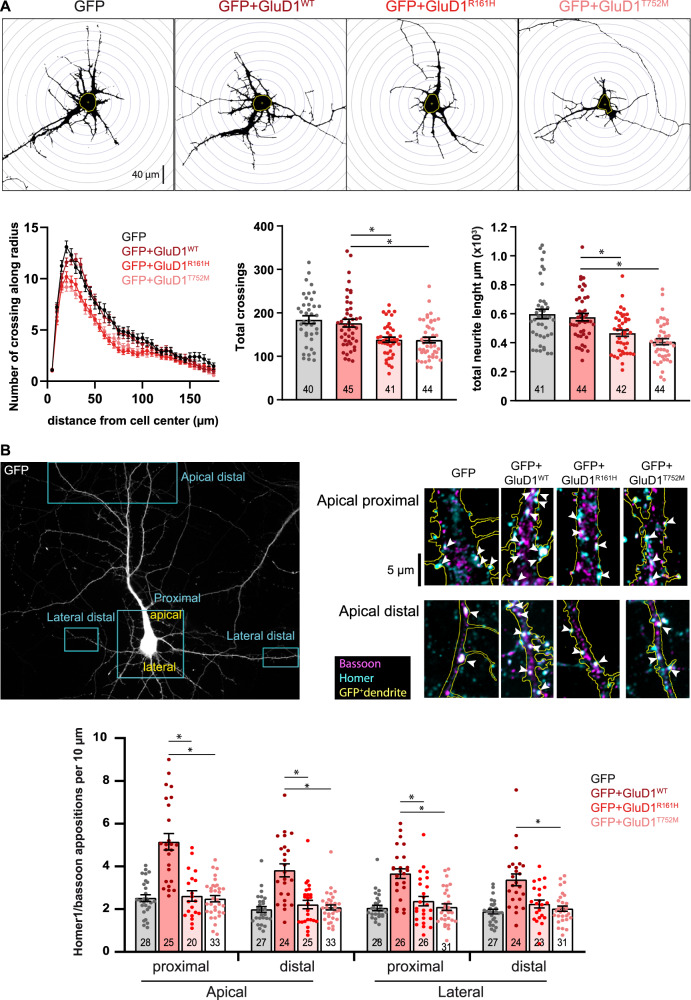
Pathophysiological impact of GluD1^R161H^ and GluD1^T752M^ mutants on neuronal morphology and synaptic density. **A** Binary images show GFP fluorescence of cultured hippocampal neurons expressing GFP alone, or GFP and indicated GluD1 variants, after plasmid transfection. Neurites crossing concentric circles centered on each neuron’s soma were counted to quantify neurite ramification. Total neurite length is the sum of all neuritic segments measured for each neuron. Graphs summarize results obtained in *n* ≥ 40 neurons from 3 cultures in each condition. **B** Squares on the GFP fluorescence picture of a pyramidal-shaped hippocampal neuron in transfected culture (*upper left*) exemplify regions where excitatory putative synapses, revealed by overlap of presynaptic Bassoon and postsynaptic Homer immunostaining on GFP positive dendrites (*upper right*), were counted. The graph shows results obtained in *n* ≥ 20 pyramidal-shaped hippocampal neurons from 3 cultures in each transfection condition indicated. *Significant differences.

Analyses of GFP-labeled neurites using the Sholl method revealed a significant reduction in total neuritic length of neurons overexpressing GluD1 mutants, as compared to control (GFP only) and GluD1^WT^-overexpressing neurons (control: 598 ± 33 µm, GluD1: 577 ± 25 µm, GluD1^R161H^: 466 ± 21 µm, GluD1^T752M^: 407 ± 21 µm; *n* = 41, 44, 42, 44 neurons, respectively, from 3 cultures in each condition; Fig. 4A). This was associated with a significantly reduced neuritic ramification of neurons transfected with GluD1^R161H^ or GluD1^T752M^ (total crossings; control: 184 ± 9, GluD1: 176 ± 9, GluD1^R161H^: 138 ± 6, GluD1^T752M^: 138 ± 6; *n* = 40, 45, 41, 44 neurons, respectively; Fig. 4A). These findings indicate that the GluD1 R^161^H and T^752^M mutations perturb neurite outgrowth and architecture.

GluD1 is present at excitatory postsynaptic sites [9, 15, 16], and promotes the formation of dendritic spines and excitatory synapses [12, 13, 29, 52]. We thus evaluated the effect of the R^161^H mutation on spine density and morphology, and found that this mutation impairs GluD1 stimulatory effects on dendritic spine formation and maturation (Supplementary information and Supplementary Fig. S10). Further analyses in hippocampal organotypic slice cultures indicated that R^161^H and T^752^M mutations also impair the enhancing effect of GluD1 on dendritic spine density in an integrated neural environment (Supplementary information and Supplementary Fig. S11). Finally, we examined the impact of the GluD1 R^161^H and T^752^M mutations on excitatory synapse density by counting overlaps of presynaptic Bassoon and postsynaptic Homer immunolabelling on GFP-expressing pyramidal-shaped neurons from hippocampal primary cell cultures. As shown in Fig. 4B, we observed a significantly higher density of putative excitatory synapses on both proximal and distal parts of apical and basal dendrites of GluD1^WT^-transfected compared to control neurons (apical proximal: 5.1 ± 0.4 vs. 2.5 ± 0.2; apical distal: 3.8 ± 0.3 vs. 2.0 ± 0.1; lateral proximal: 3.7 ± 0.2 vs. 2.0 ± 0.1; lateral distal: 3.4 ± 0.3 vs. 1.9 ± 0.1 per 10 µm dendrite of GluD1^WT^ vs. control neurons, respectively; *n* = 25, 24, 26, 24 GluD1^WT^ and *n* = 28, 27, 28, 27 control neurons, respectively), consistent with the synaptogenic function of GluD1 [12, 29, 52]. Conversely, the density of excitatory synapses on neurons overexpressing GluD1^R161H^ or GluD1^T752M^ was similar to that on control neurons (apical proximal: 2.6 ± 0.2 and 2.5 ± 0.1; apical distal: 2.2 ± 0.2 and 2.1 ± 0.1; lateral proximal: 2.4 ± 0.2 and 2.1 ± 0.1; lateral distal: 2.2 ± 0.2 and 2.0 ± 0.1 per 10 µm dendrite of GluD1^R161H^ and GluD1^T752M^ neurons, respectively; *n* = 20, 25, 26, 23 GluD1^R161H^ and *n* = 33, 33, 31, 31 GluD1^T752M^ neurons, respectively), suggesting that the role of GluD1 in excitatory synapse formation and stabilization is hampered by the R^161^H and T^752^M mutations.

These results indicate that regulation of neurite outgrowth, architecture, spine density and maturation, and excitatory synapse density are impaired by the GluD1 R^161^H and T^752^M mutations. Given the widespread distribution [15, 16] of GluD1, the R^161^H and T^752^M mutations are thus likely to affect critically the formation and function of brain networks.

## Discussion

We report association between homozygous missense variants p.Arg161His and p.Thr752Met in the *GRID1* gene, and disease phenotypes including ID, SPG, and glaucoma, in two different sibships born to consanguineous parents. Our experimental findings indicate that these *GRID1* variants impair mGlu1/5 signaling, as well as dendritic morphology and excitatory synapse density, in mouse forebrain neurons.

### Homozygous *GRID1* variants causing ID and SPG with or without glaucoma

Here, we characterize pathogenic recessive *GRID1* mutations linked to syndromic ID and SPG without or with glaucoma, a triple phenotypic association whose genetic bases had not been elucidated previously. The constant association of ID with SPG and glaucoma is rare, as only two affected families have been described [3, 4], but glaucoma has been mentioned in patients affected with SPG45 and SP75, two conditions usually comprising only ID and SPG [53, 54]. The two affected sibships reported herein share some phenotypic features and differ in others. Inter- and intra-familial phenotypic variability is well-described in inherited neurodevelopmental disorders [55] and hereditary SPG [56], and might explain some of these differences. Characterization of the full clinical spectrum of the present syndrome and elucidation of possible genotype-phenotype correlations thus await identification of additional affected individuals.

GluD1 is a postsynaptic protein widely expressed in the brain [15, 16]. While many genetic variants linked to ID concern synaptic proteins [47–49], only few SPG-linked genes [2, 57], and no glaucoma-associated genes [58, 59] identified so far encode synaptic proteins. However, several SPG-linked variants impact synapses (e.g. *AP4M1* variants altering iGluRs trafficking [60–62]), and synaptic changes appear to underlie early dysfunction of retinal ganglion cells in glaucoma [63]. Hence, the *GRID1* p.Arg161His and p.Thr752Met variants are rare examples of genetic alteration in a synaptic protein causing ID and SPG with or without glaucoma, but the existence of such mutations is consistent with synaptic impairments occurring in all three pathologies.

### The GluD1 R^161^H and T^752^M mutants impair mGlu1-5 signaling, dendrite architecture and excitatory synapses

We found that mGlu1/5 signaling via Ca^2+^ and the non-canonical ERK pathway is hampered by the GluD1 R^161^H and T^752^M mutations. Both Ca^2+^ and ERK signals are involved in neurite growth and maintenance, synapse formation and plasticity [64–68]. This suggests that part of the pathogenic impact of GluD1 R^161^H and T^752^M mutations stems from impaired signaling of the mGlu1/5-GluD1 complex. Nonetheless, additional deleterious effects of these mutations may occur due to dysregulation of yet other signaling mechanisms [10, 11, 14, 30] involving GluD1.

We also found that dendrite outgrowth, architecture, spine density and maturation, and excitatory synapse density are impaired by the GluD1 R^161^H and T^752^M mutations, despite cerebellin binding, thus trans-synaptic scaffolding, being preserved in both GluD1 mutants. This confirms that transmembrane signaling by GluD1 is essential to its role in the formation and regulation of excitatory synapses [12, 14]. Since loss of expression or function of GluD1 impairs diverse glutamatergic synapses in the forebrain, midbrain and cerebellum [9, 12–15, 29, 31], it is likely that the pathogenic effects of GluD1 R^161^H and T^752^M mutations on neuronal morphology and connectivity extend broadly in the brain.

### Implications for ID, SPG and glaucoma

GluD1 is expressed in neurons that form connections altered in ID: neurons of forebrain networks [15, 16], in SPG: pyramidal cells of the motor cortex [15, 16] and their spinal motoneuron targets [69], and in glaucoma: retinal ganglion cells [70, 71] and their targets in sensory thalamus and superior colliculus [15, 16]. This indicates that the observed cellular impact of GluD1 mutants has direct relevance to these pathologies. Indeed, dysregulation of mGlu1/5 signaling and synaptic alterations in forebrain neurons are tightly linked to ID and related neurodevelopmental disorders [46–51]. Likewise, GluD1 mutants are likely to affect the formation and maintenance of long-range pyramidal tract and optic nerve connections by altering both projection and target neurons properties. The high sensitivity of the corticospinal tract to changes in ERK signaling level [72], and the importance of mGlu1/5 for the excitability of retinal ganglion cells and their connectivity to thalamic targets [73, 74], are documented indications that GluD1 mutants can indeed contribute to corticospinal axons and optic nerve damage that cause SPG and glaucoma, respectively.

In conclusion, we report the first pathogenic variants of the *GRID1* gene in patients presenting with ID and SPG with or without glaucoma and provide evidence that these variants impair mGlu1/5 signaling, dendrite outgrowth, architecture, spine density and maturation, and synapse density. Although the present study does not exhaust the possible pathophysiological effects of GluD1^R161H^ and GluD1^T752M^ mutants, our observations demonstrate that their expression has deleterious consequences on neurons and circuits that can cause ID, SPG and glaucoma.

### Supplementary information

Supplementary Material Ung et al. GRID1/GluD1 variants in ID
