# Supplementary material for: GRID1/GluD1 homozygous variants linked to intellectual disability and spastic paraplegia impair mGlu1/5 receptor signaling and excitatory synapses
Source: Mol Psychiatry. 2024 Feb 28;29(4):1205–15. doi: 10.1038/s41380-024-02469-w (PMC11176079; doi:10.1038/s41380-024-02469-w)
Supplement: Supplementary file 1 — Supplementary Material Ung et al. GRID1/GluD1 variants in ID [file 41380_2024_2469_MOESM1_ESM.pdf]

SUPPLEMENTARY INFORMATION – Ung *et al***.** ***GRID1*****/GluD1 variants in ID**

- Supplementary Materials and Methods………………………………………… p1
- Supplementary Table: Antibodies………………………………….………… p10
- Supplementary Results
  - o Clinical description of Family B………………………………..………… p11
  - o Alteration of D-serine and glycine effects by the $R^{161}H$ and $T^{752}M$ mutations… p11
  - o The R161H and T752M mutations do not hamper cerebellin binding………… P12
  - o Impact of $GluD1^{R161H}$ and $GluD1^{T752M}$ on dendritic spines……......................... p12
- Supplementary References……………………... p14
- Supplementary Figure Legends………………... p17
- Supplementary Figures 1-11……………………………………………………. P20

Supplementary Materials and Methods

Patients

Written informed consent for genetic analysis was obtained from all participants or their legal guardians according to the Declaration of Helsinki and following Institutional Review Board (IRB)-approved protocols in the Centre Hospitalier Universitaire de Tours medical center (Family A) and the Hadassah Medical Center (Family B).

Animals

Animal breeding and euthanasia were performed in accordance to European Communities Council Directive 86/609/062. *Grid1* KO mice (Gao *et al.*, 2007; gift from Jian Zuo, Memphis, TE, USA) were genotyped as described (Hepp *et al.*, 2015). Homozygous *Grid1* KO mouse embryos were obtained from breeding heterozygous parents. Wild-type (wt) mice were purchased from Janvier Labs. All mice had C57BL/6 background.

Genome wide-linkage analysis and whole exome sequencing

Genomic DNA samples were extracted from peripheral blood following standard protocols. Genotyping of Family A (three affected children, one healthy child and both consanguineous parents) was performed on Genechip® human 250K NspI array (Affymetrix) according to manufacturer's instructions. Briefly 250 ng of genomic DNA were restricted with NspI. NspI adaptators were then ligated to restricted fragments followed by PCR using universal primer PCR002. PCR fragments were purified and 90 µg were used for fragmentation and end-labelling with biotin using Terminal Transferase. Labelled targets were then hybridized overnight to Genechip® human 250K NspI array (Affymetrix) at 49°C. Chips were washed on the fluidic station FS450 following specific protocols (Affymetrix) and scanned using the GCS3000 7G. The image was then analyzed with GCOS software to obtain raw data (CEL files). Genotypes were called by the Affymetrix GType software using Dynamic Model (DM) and Bayesian Robust Linear Model with Mahalanobis (BRLMM) mapping algorithms. Homozygosity regions were obtained using MERLIN software assuming a recessive model with complete penetrance (disease allele frequency of 0.0001).

WES study was performed using Agilent SureSelect Human All Exon kit (V2; Agilent technologies). Genomic DNA was captured with biotinylated oligonucleotides probes library (Agilent technologies), followed by paired-end 75 bases massive parallel sequencing on Illumina HiSEQ 2000. Image analysis and base calling were performed using the Illumina Real-Time Analysis Pipeline version 1.14 with default parameters. Sequencing data was analyzed according to the Illumina pipeline (CASAVA1.7) and aligned with the Human reference genome (hg19) using the ELANDv2 algorithm. Genetic variation

annotation was performed with the IntegraGen in-house pipeline (IntegraGen). Filtering was performed using Eris software (IntegraGen) with an autosomal recessive hypothesis. Variants with minor allele frequency (MAF) >1% in either the 1000 Genomes Project, the EXAC, or the gnomAD databases were excluded. Genetic segregation of the candidate variant with the disease in Family A was confirmed by Sanger sequencing of *GRID1* exon 3.

For Family B, DNA sample of the proband was shipped to Otogenetics, USA (CLIA lab). ~50 Mb of genomic DNA were captured on HiSeq 2500. Fragments were read 100-125 bp, paired end. The sample was uploaded onto DNAnexus software and 71.5 million reads were aligned to the reference human genome (Hg19) (Mean on target coverage, X118). Variants which were low covered, off target (>6bp from splice site), synonymous, heterozygous, predicted as benign, MAF>0.5% on ExAC and MAF>4% in the Hadassah in-house dbSNP were removed. Thirty-one homozygous variants survived this filtering.

Molecular modeling of GluD1 mutants structure

The protein was generated using Rat GluD1 receptor in complex with 7-chloro-kynurenate and calcium ions (PDB codes: 6KSS and 6KSP) as structure templates (Burada *et al.*, 2020). The system with proteins and ligands was prepared in the CHARMM-GUI web server (Jo *et al.*, 2008) in order to generate a membrane around the protein and solvate with water and ions. A heterogeneous membrane made of POPC was chosen, and a TIP3 water model with NaCl (0.15 M) counter ions was chosen for the solvation. The system was typed with a CHARMM36m force field, and NAMD protocol was used. The system was equilibrated through six constrained simulations for a total of 500 ps by gradually diminishing the force constraints at each steps. The following constraints were applied (each value represents an equilibration step): protein backbone (5/2.5/1/0.5/0.1 kcal/mol), protein side chains (5/2.5/1.25/0.5/0.25/0.05 kcal/mol), lipid heads (5/5/2/1/0.2/0 kcal/mol), and dihedral bonds (500/200/100/100/50/0 kcal/mol). Then, a production dynamic of 10 ns was carried out in *NPT* conditions at 303.15 K without any constraints.

Mutant models were generated using Built Mutant protocol from Discovery Studio 2019. A set of 100 structures was created and ranked for their Dope score. The best model was then minimized using Adopted Basis Newton-Raphson algorithm (a Newton-Raphson algorithm applied to a subspace of the coordinate vector spanned by the displacement coordinates of the last positions) until a RMS gradient of 0.001 was obtained.

Molecular docking experiments of D-Serine, glycine and kynurenic acid at the active site were performed as described (Ducassou *et al.*, 2015, Dhers *et al.*, 2017), using default parameters from CDocker (Wu *et al.*, 2003) with Discovery Studio 2020 and a sphere radius of 10 Å in rigid mode. Flex Dock (Discovery Studio) was used for ligand-protein flexible docking. Dockings were performed on minimized structure of receptor before dynamics experiments.

Recombinant protein production and Bio-Layer Interferometry (BLI) measurements of GluD1-cerebellin interaction

Mouse wild-type (WT) full-length (FL) Cerebellin-1 ($Cbln1_{FL}$; Q22-L193), fused to a C-terminal Avitag and His6 tag, was subcloned into the pHR-CMV-TetO2 vector. A polyclonal stable HEK293T cell line expressing $Cbln1_{FL}$ was constructed using lentiviral transduction as previously described (Elegheert *et al.*, 2018; Behiels and Elegheert, 2021), and was subsequently expanded into large-volume roller bottle format. Secreted $Cbln1_{FL}$ was purified from conditioned expression media using Ni^{2+}-immobilized metal affinity chromatography (IMAC) followed by size exclusion chromatography (SEC). The fractions presenting pure protein were pooled, concentrated to 16.3 mg/mL (corresponding to 120 µM for the native hexameric protein) and stored in 20 mM HEPES pH 7.4, 150 mM NaCl, 3 mM CaCl2, 0.005% Tween-20.

Mouse $GluD1_{ATD\text{-}LBD}$-Fc was constructed by fusing the GluD1 amino-terminal domain and ligand-binding domain (ATD-LBD; D21-T814; with a GT linker between K547 and P664 to remove the M1-M2-M3 transmembrane helices) with the fragment crystallizable (Fc) region of human IgG1. Mutations $R^{161}H$ or $T^{752}M$ were introduced onto this scaffold. WT, $R^{161}H$ or $T^{752}M$ $GluD1_{ATD\text{-}LBD}$-Fc proteins were transiently expressed in HEK293T cells, and conditioned expression media containing the secreted proteins were collected 48-60h after transfection.

The direct interaction between $Cbln1_{FL}$ and $GluD1_{ATD\text{-}LBD}$-Fc (WT, $R^{161}H$ and $T^{752}M$) was measured using a BLI Octet® R8 (Sartorius). Octet® AHC biosensors (Sartorius) were coated by dipping them into conditioned expression media containing $GluD1_{ATD\text{-}LBD}$-Fc (WT, $R^{161}H$ and $T^{752}M$) at 25 °C to a final optical thickness of 1.1 nm. Assays were performed at 25 °C in a volume of 200 µL in 20 mM HEPES pH 7.4, 150 mM NaCl, 3 mM CaCl2, 0.1% BSA and 0.005% TWEEN 20. A duplicate set of sensors without protein was used as a background binding control. Association of $Cbln1_{FL}$ (5000, 2500, 1250, 625, 312.5, 156.25, and 78.125 nM) to coated and uncoated reference sensors was measured over 300 s and dissociation over 1600 s after switching to $Cbln1_{FL}$-free buffer. Data analysis on the Octet® R8 instrument was performed using a double reference subtraction (sample and sensor references) in the Octet® Analysis studio software (version 13), which accounts for nonspecific binding, background, and signal drift and minimizes well-based and sensor variability. K_D values were fitted locally and separately using a 2:1 heterogeneous binding model to account for avidity effects stemming from the multivalent nature of $Cbln1_{FL}$ (dimer of trimers) and $GluD1_{ATD\text{-}LBD}$-Fc (dimer). Finally, processed raw data and fitted curves were exported from Octet® Analysis studio software and plotted in GraphPad Prism (Version 10.0.2). The K_{a1}/K_{dis1} and K_{a2}/K_{dis2} association/dissociation values for the individual concentrations were plotted onto iso-affinity graphs for easier visualization.

Plasmids and viruses

The following plasmids encoding mouse wild-type GluD1 ($GluD1^{WT}$, Hepp *et al.* 2015), mouse GluD1 variants, rat mGlu1a, GFP or tdTomato were used for expression in HEK293 cells, neurons or *Xenopus laevis* oocytes.

HEK293 cells: pcDNA3.1-HA-$GluD1^{R161H}$, pcDNA3.1-HA-$GluD1^{T752M}$, pRK5-HA-mGlu1a-Venus

Neuronal primary cultures: pmaxGFP (Lonza), pCMX-GFP (Umesono 1991; Drobac *et al.*, 2010), pCMV-HA-$GluD1^{WT}$, pCMV-HA-$GluD1^{R161H}$

HEK293 cells, neuronal primary cultures and organotypic slice cultures: pcDNA3.1-HA-$GluD1^{WT}$, pDEST26-$GluD1^{WT}$, pDEST26-$GluD1^{R161H}$, pDEST26-$GluD1^{T752M}$

Organotypic slice cultures: pCAG-tdTomato, generous gift of R. Tsien (UC San Diego, CA)

Xenopus oocytes: pSGEM-$GluD1^{F655A}$, pSGEM-$GluD1^{F655A+R161H}$, pSGEM-$GluD1^{F655A+T752M}$, pSGEM-Δser-$GluD1^{F655A}$.

The hemagglutinin (HA) epitope YPYDVPDYA was inserted just after the predicted signal peptides of GluD1 and mGlu1a, this latter additionally comprising the Venus GFP variant fused to its C-terminus. Plasmids pRK5-HA-mGlu1a-Venus, pDEST26-$GluD1^{WT}$, and pcDNA3.1-HA-$GluD1^{WT}$ have been described previously (Perroy *et al.*, 2008, Benamer *et al.*, 2018). The $R^{161}H$ and $T^{752}M$ mutations were introduced in GluD1 through site-directed mutagenesis using the QuikChange II XL kit (Agilent Technologies). For generating pCMV-HA-$GluD1^{WT}$, the full length coding sequence of the *Grid1* cDNA (Genbank accession number: BC167177) was PCR amplified from clone A230054J23 (Mus musculus adult male hypothalamus cDNA, RIKEN full-length enriched library, Refseq AK138279, Source BioScience) and inserted into the pCMV-HA-C plasmid (Clontech). A stop codon was then added at the end of the *Grid1* coding sequence upstream of the plasmidic HA tag, before inserting a HA tag after the predicted signal peptide. For expression in *Xenopus* oocytes, the $GluD1^{WT}$ cDNA was subcloned into pSGEM (Villmann *et al.*, 1999) and all point mutations were introduced by IVA cloning (García-Nafría

et al., 2016). The constitutively open channel GluD1^{F655A} mutant retains modulation by D-serine and glycine (Yadav *et al.*, 2011). The Δser-GluD1^{F655A} additionally carries R^{526}K and D^{742}A mutations that disrupt D-serine and glycine binding to the LBD (Hansen et al., 2009). All constructs were verified with DNA sequencing.

Recombinant viruses were used for transduction of neurons in primary cortical cell culture. Lentiviruses LV-PGK-GluD1^{WT}-ires-GFP, LV-PGK-GluD1^{R161H}-ires-GFP and LV-PGK-GluD1^{T752M}-ires-GFP were generated exactly as described (Benamer *et al.*, 2018) for co-expression of GluD1^{WT}/GluD1^{R161H}/GluD1^{T752M} and GFP driven by the PGK promoter. Recombinant lenti pseudo-virions were produced at the Viral Vector and Gene Transfer facility of the Necker Institute (IFR94, Paris, France). Recombinant Sindbis virus Sin-Twitch-2B encoding the ratiometric calcium sensor Twitch-2B (Thestrup et al. 2014) was produced as described (Gervasi et al. 2007).

Electrophysiology on *Xenopus laevis* oocytes

To obtain oocytes, parts of the ovaries were surgically removed from *Xenopus laevis* (Xenopus 1, Dexter, MI, USA) anaesthetised with 3-aminobenzoic acid ethylester (1.5 g/L, Sigma, Taufkirchen, Germany). To remove the follicular cell layer, the ovary clippings were digested with collagenase type I (4 mg/mL, Worthington, Lakewood, NJ, USA) in Ca^{2+}-free Barth's solution (88 mM NaCl, 1.1 mM KCl, 2.4 mM $NaHCO_3$, 0.8 mM $MgSO_4$, 15 mM HEPES-NaOH, pH 7.6) for 1.5–2 h at 20 °C and then washed with Barth's solution (88 mM NaCl, 1.1 mM KCl, 2.4 mM $NaHCO_3$, 0.3 mM $Ca(NO_3)_2$, 0.4 mM $CaCl_2$, 0.8 mM $MgSO_4$, 15 mM HEPES-NaOH, pH 7.6) to stop the digestion. Complementary RNA (cRNA) for injection into oocytes was synthesized from 1 µg linearized plasmid DNA with the mMESSAGE mMACHINE T7 *in vitro* transcription kit (Ambion, Austin, TX, USA). Defolliculated oocytes of stages V and VI were manually selected, maintained at 16 °C in Barth's solution supplemented with gentamicin (100 µg/mL), streptomycin (40 µg/mL), and penicillin (63 µg/mL), and injected with 10 fmol (11 ng) of cRNA using a nanoliter injector (WPI, Sarasota, FL, USA). Four to five days after cRNA injection, current responses were recorded under voltage clamp at –70 mV with a Turbo Tec-10CX amplifier (npi electronic, Tamm, Germany) controlled by Patchmaster software (HEKA, Lambrecht, Germany). Currents were filtered with a 20 Hz low-pass filter and then digitised with a sampling rate of 50 Hz. Recording electrodes with resistances of 0.5–1.5 MΩ were pulled from borosilicate glass (Science Products GmbH, Hofheim, Germany) with an L/M-3P-A vertical pipette puller (List-Medical) and filled with 3 M KCl. Recordings were performed in a 50 µL chamber under constant superfusion at a flow rate of 3–5 mL/min. To assess the background current without any Na^+ flux, the oocyte was superfused with a Na^+-free solution containing the impermeable cation N-methyl-D-glucamine (NMDG; 115 mM NMDG-Cl, 2.5 mM KCl, 1.8 mM $BaCl_2$, 10 mM HEPES, pH 7.2) until a stable baseline was reached. The recording was started, and after 10 s, Na^+-containing Ba^{2+} Ringer's solution (115 mM NaCl, 2.5 mM KCl, 1.8 mM $BaCl_2$, 10 mM HEPES-NaOH, pH 7.2) was applied for 30 s to determine the spontaneous Na^+ current through permanently open channels. Then, the substance to be tested for its effect on the spontaneous current (3 mM D-Ser, 3 mM Gly or 100 µM pentamidine in Ba^{2+} Ringer's solution) was applied for 20 s, then washed out for 20 s with Ba^{2+} Ringer's solution and finally for 40 s with Na^+-free NMDG solution to check for reversibility.

HEK293T cell culture and transfection

HEK293T cells (ATCC Number: CRL-3216, authenticated using Short Tandem Repeat analysis by the ATCC cell authentication service, mycoplasma-free) were cultured in Dulbecco's modified Eagle's medium (DMEM) supplemented with 10% fetal bovine serum, 100 U/ml penicillin and 100 µg/ml streptomycin (Life Technologies). For immunostaining or cerebellin binding experiments, cells were seeded at 8.10^5 cells per well on glass coverslips coated with poly D-lysine (Sigma Aldrich P7280) and

cultured in 12-well plates. For membrane protein isolation and immunoprecipitation experiments, cells were seeded in 10 cm dishes coated with poly D-lysine at a density of 2.10^6 cells/dish. Transient plasmid transfection was performed the next day using the calcium phosphate precipitation method (6 µg plasmid per 12 well-plate or per dish) or using Lipofectamine 2000 (Invitrogen, 2,5 µg plasmid and 6µL reagent per 6 well-plate). Plasmids encoding mGlu1-YFP and GluD1 were mixed at a ratio 1:1 for co-transfection. Culture medium was renewed 6 h after transfection, and cells cultured overnight.

Immunostaining on HEK cells

Transfected HEK cells were fixed with 4% paraformaldehyde in 0.1 M sodium phosphate buffer (PB) during 20 min, and then washed with Dulbecco's phosphate-buffered saline (D-PBS). All the procedure was performed at room temperature. After fixation, cells were incubated in PBS containing fish skin gelatin (2g/l) and Triton X100 0,25% (PBS-GT) for 1 hour. Triton X100 was omitted from incubation medium (PBS-G) when cells were not permeabilized. Next, cells were incubated for 2 to 4 hours with primary antibodies (see **Suppl. Table** for antibodies) diluted in PBS-GT/PBS-G, washed 3 times 15 minutes with PBS, and incubated with secondary antibodies (**Suppl. Table**) and DAPI nuclear stain (300 nM, Invitrogen) diluted in PBS-GT for 2 hours. After PBS washes, samples were mounted on glass slides using Fluoromount-G (Biovalley 0100-01), and images were acquired using an epifluorescence microscope (DMR, Leica), or a confocal microscope (SP5, Leica).

Cerebellin binding on HEK cells

HEK cells expressing GluD1, $GluD1^{R161H}$ or $GluD1^{T752M}$ were incubated for 1 hour at 35 °C in culture medium containing 20 µg/ml recombinant human HA-tagged Cerebellin 1 (Cbln1, Biotechne 6934-CB-025). After two washes with ice-cold PBS, cells were fixed and processed for immunostaining as described above using rabbit anti-GluD1 and mouse anti-HA primary antibodies (**Suppl. Table**).

Isolation of membrane proteins from HEK cells and western blotting

Total membrane proteins were extracted from HEK cells expressing $HA\text{-}GluD1^{WT}$, $HA\text{-}GluD1^{R161H}$ or $HA\text{-}GluD1^{T752M}$ using the MEM-Per™ Plus Membrane Protein extraction kit (Thermoscientific) according to manufacturer's protocol. Proteins lysates were separated on 4-20% Mini-PROTEAN® TGX Stain-Free Precast electrophoresis Gels (Bio-Rad) and transferred using Trans Blot Turbo system (Bio-Rad) on nitrocellulose membranes (Bio-Rad). Membranes were then incubated in blocking buffer with 5% milk in a mixture of Tris-buffered saline and Tween 0,002% (TBST, Fisher) for 1 hour at room temperature. Next, membranes were incubated with rat anti-HA antibody (**Suppl. Table**) overnight at 4°C in 5% milk diluted in TBST. After three washes of 10 min, membranes were incubated in 5% milk-TBST with secondary anti-rabbit and anti-beta-actin antibodies conjugated with horseradish peroxydase (HRP, **Suppl. Table**) for 45 min. HRP was revealed through chemiluminescence using Clarity™ Western ECL substrate (Bio-rad), visualized on a ChemiDoc™ Touch imaging system (Bio-rad), and quantified using the ImageJ software (U.S. National Institutes of Health, Bethesda, MD, USA; http://rsbweb.nih.gov/ij/).

Immunoprecipitation from HEK cells

HEK cells co-expressing HA-mGlu1a-Venus and GluD1, $GluD1^{R161H}$, or $GluD1^{T752M}$ were washed twice with ice cold PBS and lysed in 500 µl lysis buffer containing 50mM Tris-HCl pH 7.5, 150mM NaCl, 1% Nonidet P40, 0.5% sodium deoxycholate, and protease inhibitor (Complete Ultra Tablets, Roche) according to manufacturer's instructions. The whole immunoprecipitation procedure was carried out at 4 °C. Lysates were centrifuged 13000g for 15 min and protein concentration was determined in the supernatant by the Bradford's method using BSA as standard. Supernatants were then pre-cleared with Protein A Plus Agarose beads (Pierce). Specific immunoprecipitation were performed overnight by incubating 250 µg proteins of the precleared lysates with specific antibodies or control rabbit anti-mouse

antibodies (**Suppl. Table**). Protein complexes bound to rabbit anti-GluD1 antibodies were precipitated with Protein A Plus agarose beads for 4 h. Protein complexes bound to mouse anti-HA antibodies were precipitated with beads coupled to rabbit anti-mouse antibodies. Precipitates were washed twice with lysis buffer, twice with 50 mM Tris Hcl pH7.5, 500 mM Nacl, 0.1% Nonidet P40, 0.05% Sodium deoxycholate and once with 50 mM Tris Hcl 0.1% Nonidet P40, 0.05% Sodium deoxycholate. Proteins were eluted from the beads with 30 µl LDS sample buffer (Invitrogen), separated on 4-15 % polyacrylamide gels (Biorad), and transferred onto nitrocellulose membranes. Western blots were carried out using standard protocols and antibodies listed in **Suppl. Table**. Detection was performed with the Odyssey detection system (LI-COR Bioscience) using secondary anti-IgG antibodies coupled to infrared dyes (**Suppl. Table**). Band intensity was determined using ImageJ.

Primary cortical or hippocampal cell cultures

All components for cell cultures were from Thermo Fisher Scientific unless otherwise stated. Primary cortical or hippocampal cell cultures were prepared essentially as described (Ung *et al*., 2018) from E17-E18 *Grid1*$^{-/-}$ or *Grid1*$^{+/+}$ mice embryos, respectively. Cortices and hippocampi were dissected in ice cold PBS containing 100 U/ml penicillin and 100 µg/ml streptomycin, and kept in Hibernate E medium supplemented with 2% B27, while genotyping using the Phire Animal Tissue Direct PCR Kit (Thermo Fisher, for primers see Hepp *et al*., 2015). Tissues were pooled, dissociated with papain (Worthington). Tissues were then triturated in DMEM-F12 containing 10 % heat inactivated fetal calf serum and cells transferred to a new tube and centrifuged 250 g for 4 minutes. Cells were resuspended in Neurobasal medium supplemented with 2% B27, 0.5 mM glutamax (complete Neurobasal medium), and counted. Hippocampal cells were seeded at a density of $6x10^4$ cells/500 µl medium per well on glass coverslips coated with poly D-lysine and laminin (Sigma Aldrich) in 24 well plates. For Ca^{2+} imaging, cortical cells were cultured as described for hippocampal cells, whereas for western blot analyses, cortical cells were plated at 10^6 cells per dish on 35 mm culture dishes coated with poly D-lysine and laminin. Cells were grown at 35°C, under 5% CO2 atmosphere. Half of the medium was changed every 3 to 4 days.

Viral transduction of cortical cell cultures and test of mGlu1/5 signaling

At 10 days *in vitro* (DIV), cultures were transduced with LV-PGK-GluD1-ires-GFP, LV-PGK-GluD1^{R161H}-ires-GFP, or LV-PGK-GluD1^{T752M}-ires-GFP recombinant pseudo-virions at a density of infection of 1:1, and then cultured for 4 to 7 additional days.

For measurements of lentiviral transduction efficiency, cultures were next fixed and processed for DAPI staining and immunolabelling (see **Suppl. Table**) as described above for HEK cells.

For test of mGlu1/5 signaling via the ERK pathway, cultures were next rinsed once with warm HBSS-TTX-APV medium containing 2 mM Ca2+, 1 mM Mg2+, 300 nM TTX (Latoxan), and 50 µM of the NMDAR antagonist APV (Hello Bio). Cells were then incubated for 1 hour at 35° in HBSS-TTX-APV. The medium was next removed, and cells were incubated for 5 minutes at 35°C in HBSS-TTX-APV medium, in the presence or absence of the mGlu1/5 agonist RS-3,5-dihydroxyphenylglycine 2 (DHPG, 100 µM, Hello Bio). Cells were then lysed in ice cold 50 mM Tris-HCl pH 7.5, 150 mM NaCl, 1% Nonidet P40, 0.5% sodium deoxycholate, supplemented with protease (Complete Ultra Tablets, Roche) and phosphatase inhibitors (PhoStop, Roche) according to manufacturer's instructions. Protein concentration was determined using the Bradford's method with BSA as standard. Proteins (10 µg/lane) were separated by SDS-PAGE, transferred onto nitrocellulose sheets, and western blots were carried out using standard protocols. Primary antibodies and secondary anti-IgG antibodies coupled to infrared dyes are listed in **Suppl. Table**. Detection was performed with the LI-COR Odyssey detection system. Band intensity was determined using the ImageJ software.

For live imaging of mGlu1/5-dependent Ca^{2+} signaling, 0.5µl of the recombinant Sin-Twitch-2B pseudo-virion solution (10^4 i.p.) was added per well onto cortical neurons cultured on glass coverslips. The next day, glass coverslips were transferred to a recording chamber and neurons were perfused with a solution containing (in mM): 110 NaCl, 5.4 KCl, 1.8 CaCl2, 0.8 MgCl2, 10 HEPES, 10 D-Glucose at a rate of 2 ml/min at room temperature. The mGlu1/5 agonist S-3,5-dihydroxyphenylglycine 2 (S-DHPG, 50 µM, Hello Bio) was applied through the bath perfusion. Two photon image stacks were obtained every 15 seconds with a custom-built 2-photon laser scanning microscope as described (Bonnot *et al.*, 2014) using a 20x (0.5 NA) water immersion objective. Image acquisition was performed using the Matlab routine ScanImage (Pologruto *et al.*, 2003). The ratiometric Ca^{2+} sensor Twitch-2B is based on the fluorescent CFP/YFP variants of GFP as donor/acceptor pair (Thestrup et al. 2014). Two-photon excitation was performed at 850 nm for CFP with a tunable femtosecond Ti:sapphire pulsed laser (MaiTai HP; Spectra Physics, Ellicot City, MD, USA). CFP and YFP fluorescence were collected on two independent photomultipliers. Images were analyzed using custom written macros in ImageJ and the emission ratio YFP/CFP calculated for regions of interest (ROI) drawn around the somata of Twitch-2B-expressing neurons. Fluorescence intensity variations in a given ROI was expressed as the ratio $\Delta R/R_0$, were $\Delta R = R - R_0$. R is the YFP/CFP ratio in a ROI at a given time point, and R_0 corresponds to the YFP/CFP ratio in the same ROI during control baseline prior to drug application.

Plasmid transfection of hippocampal cell cultures and analyses of neurites and excitatory synapses

Cultures were transfected at DIV4 for morphometric analyses of dendrites, and at DIV11-DIV13 for analyses of spine morphology and synapse counting. Cells were transfected with plasmid pCMX-GFP alone or in combination with pDEST26-GluD1^{WT}, pDEST26-GluD1^{R161H}, or pDEST26-GluD1^{T752M} (1:1 ratio) using Lipofectamine 2000 (Invitrogen). Plasmids pmaxGFP, pCMV-HA-GluD1^{WT} and pCMV-HA-GluD1^{R161H} were used instead of above plasmids for experiments shown in **Figures S3 and S8**. Lipofectamine (1 µl/well) and plasmids (500 ng/well) were diluted in Neurobasal medium (100 µl/well). Prior to transfection, 300 µl medium was collected from each well and diluted by half with fresh complete Neurobasal medium. This conditioned medium was kept in the incubator for the duration of the transfection. Next, 200 µl complement-free Neurobasal medium and 100 µl of the lipofectamine-DNA solution was added in each well. After 2 h incubation at 35°C, cells were washed twice with complete Neurobasal medium before adding 500 µl conditioned medium. Cells were then incubated for 48 h before fixation. Cells were fixed with 4 % paraformaldehyde for 20-30 min, permeabilized with PBS-GT unless otherwise stated, and processed for immunolabelling and DAPI staining as described above for HEK cells. Immunolabelling of co-transfected cultures using chicken anti GFP, and rabbit anti-GluD1 or rat anti-HA primary antibodies (**Suppl. Table**), demonstrated that more than 96 % of GFP-expressing neurons also over-expressed either GluD1^{WT}, GluD1^{R161H}, GluD1^{T752M}, HA-GluD1^{WT}, HA-GluD1^{R161H}, or HA-GluD1^{T752M}.

For morphometric analyses of dendrites, images of isolated GFP-expressing neurons were acquired with an epifluorescence microscope (DMR, Leica). The Sholl analysis was performed upon conversion to binary images using the SNT module of ImageJ/Fiji software (https://imagej.nih.gov/ij/, Schindelin *et al.*, 2012; Schneider *et al.*, 2012; Ferreira *et al.*, 2014). An ROI delimiting the soma was used to define the cell center from which concentric circles of 20 pixels (5 µm) apart were drawn on a radius of 700 pixels. For each cell, the number of neurites crossing along the radius and the total crossings were determined. The total length of neurites per cell was manually determined using the segmented line tool of ImageJ/Fiji. Lines were converted to ROIs to determine the length of all the segment per cells in order to sum them up. Neurites extending beyond the field were not included.

For analyses of dendritic spine morphology, images of isolated GFP-expressing spiny dendrites were acquired by confocal microscopy (TCS SP8-STED, Leica) using a 63X objective with a zoom of 2 and were z-sectioned at 0.3 µm increments. Morphological analysis of the GFP-labelled spines was performed manually according to Zagrebelsky *et al.* (2005), based on measurements of spine length and of the ratio between neck and head diameters of the spine. We distinguished immature spines comprising both long thin (length: 1<x<3 µm, head/neck diameter<2) and filopodia-shaped (length >3 µm) spines, versus mature spines comprising both mushroom-shaped (length: 1<x<3 µm, head/neck diameter>2) and stubby (length<1 µm) spines.

For synapse counting, cells were labelled using primary antibodies: chicken anti GFP, mouse anti Bassoon, rabbit anti Homer1, and secondary antibodies: goat anti-chicken Alexa 488, goat anti-mouse-RRX, goat anti-rabbit-Alexa 647 (**Suppl. Table**). Images were acquired with a DMR Leica epifluorescence microscope. The density of glutamatergic synapses was measured by counting manually Homer1/Bassoon co-labelled spots present on merged images of GFP positive dendrites processed with ImageJ/Fiji. Only spiny neurons exhibiting a pyramidal cell-like morphology with pyramidal-shaped soma and prominent apical dendrite were analysed.

Organotypic hippocampal slice culture, single cell electroporation and spine counting

Organotypic hippocampal slice cultures were prepared from postnatal day 5-6 C57Bl6/J mice (Stoppini *et al.*, 1991). Animals were quickly decapitated and brains placed in ice-cold Gey's balanced salt solution under sterile conditions. Hippocampi were dissected and coronal slices (350 µm) were cut using a tissue chopper (McIlwain) and incubated at 35 °C with serum-containing medium on Millicell culture inserts (CM, Millipore). The medium was replaced every 2–3 days. After 6 days in culture, organotypic slices were transferred to an artificial cerebrospinal fluid (ACSF) containing (in mM): 130 NaCl, 2.5 KCl, 2.2 CaCl2, 1.5 MgCl2, 10 D-glucose, 10 HEPES (pH 7.35, osmolarity adjusted to 300 mOsm). CA1 pyramidal cells were processed for single-cell electroporation at DIV7 using glass micropipettes containing plasmids encoding tdTomato (6 ng.µL–1) alone or together with plasmids encoding HA-GluD1^{WT}, GluD1^{WT}, GluD1^{R161H} or GluD1^{T752M} (24 ng.µL–1). Micropipettes were pulled from 1 mm borosilicate capillaries (Harvard Apparatus) with a vertical puller (Narishige). Electroporation was performed by applying 4 square pulses of negative voltage (–2.5 V, 25 ms duration) at 1 Hz, then the pipette was gently removed. 10–20 neurons were electroporated per slice, and slices were placed back in the incubator for 4 days before being processed for confocal imaging.

For visualization of recombinant HA-GluD1^{WT} and dendritic spine counting in CA1 neurons expressing tdTomato, organotypic slices were fixed with 4% paraformaldehyde- 4% sucrose in PBS for >4 h before the permeabilization of membranes with 0.25% Triton in PBS. Slices were subsequently incubated with a rat anti-HA antibody followed by a donkey anti-rat Alexa 488 antibody (**Suppl. Table**). Images were acquired on a Leica DM6 CFS TCS SP8 microscope using a 63 × /1.4 NA oil objective and a pinhole opened to 1 time the Airy disk. Images with pixel size of 70 nm were acquired at a scanning frequency of 400 Hz. The vertical step size was set at 0.3 µm. The number of spines per unit dendrite length of tdTomato-positive cells was calculated manually using Image J.

Statistical analyses

All experiments were repeated at least three times, and GraphPad prism6 software (Instat) was used for statistical analyses and graphical representations. When d'Agostino-Pearson normality tests were successfully passed, we conducted parametric test using One-way ANOVA. Then, Tukey's post hoc method was used to determine statistical significance in multiple comparisons and to reveal the contribution of the genotype in the variability between each test. For samples that did not pass the

normality test, we used Kruskal-Wallis method followed by Dunn's post hoc test. Results are given as mean ± standard error of the mean. Differences were considered significant if $p<0.05$.

<u>Supplementary Table</u>: Antibodies

Primary Antibodies	*reference*	*Immuno-precipitation*	*Immuno-fluorescence*	*Western blot*
rabbit anti-GluD1	*Hepp et al. 2015*	*7 µg/250µg prot*	*1/10,000*	*1/10,000*
rabbit Anti-Mouse IgG	*Jackson Immunoresearch 315-005-003*	*2µg/2µg mouse anti-HA*		
mouse anti-HA	*Biolegend clone 16B12*	*2 µg/250µg prot*	*1/2000*	*1/5000*
rabbit anti-HA	*Clontech Takara Bio 631207*		*1/500*	*1/1000*
rat anti-HA	*Roche, clone BMG 3F10*		*1/100*	
Chicken anti-GFP	*Aves GFP-1020*		*1/1000*	
Rabbit anti-GFP	*Chromtek PABG1*		*1/1000*	
Mouse anti MAP2	*Sigma M9942*		*1/1000*	
Rabbit anti phosphoERK1/2	*Cell Signaling 4370S*			*1/2000*
Mouse anti ERK1/2	*Cell Signaling 4696S*			*1/2000*
Mouse anti-beta-actin HRP-conjugated	*Sigma-Aldrich A3854*			*1/50000*
Secondary Antibodies				
Goat anti-rabbit HRP-conjugated	*Promega W4011*			*1/2500*
Goat anti-Mouse IgG DyLight 680	*Thermo Fisher 35519*			*1/5000*
Goat anti-Rabbit IgG DyLight 800	*Thermo Fisher SA5-10036*			*1/5000*
Donkey anti-rabbit Alexa Fluor 488	*Thermo Fisher A21206*		*1/2000*	
Donkey anti-rabbit Alexa Fluor 594	*Interchim FP-SD5115*		*1/500*	
Donkey anti-rat Alexa Fluor 488	*Thermo Fisher 712-545-153*		*1/200*	
Goat anti-Rabbit IgG Alexa Fluor 555	*Thermo Fisher A21430*		*1/2000*	
Goat anti-Mouse IgG Alexa Fluor 488	*Thermo Fisher A11029*		*1/2000*	
Goat anti-Mouse IgG Alexa Fluor 555	*Thermo Fisher A21422*		*1/2000*	
Goat anti-Mouse IgG Alexa Fluor 647	*Thermo Fisher A21235*		*1/2000*	
Goat anti-Chicken IgY Alexa Fluor 488	*Thermo Fisher A11039*		*1/2000*	

Supplementary Results

Clinical description of Family B

The proband (designated **Patient 4**), a 6 years and 3 months old girl, presented for evaluation due to intellectual disability, unique facial features, brachycephaly, recurrent episodes of hypereosinophilia and additional findings.

She is the seventh of seven children born to consanguineous (first and second degree cousin) parents of Arab-Muslim descent. She was reported to have had intrauterine growth retardation (IUGR), and was born at term (38 weeks of gestation), birth weight of 1800 grams. The parents reported of failure to thrive, hypotonia and significantly delayed acquisition of developmental milestones: she turned over at 8 months, sat up at 12 months, and began walking at the age of 2.5 years. She began talking at 5 years of age, and at 6 years was reported to have a vocabulary of ~20 words. Socially, she did not interact with her peers.

Upon physical examination (at 6 years of age), she was alert and vital, in no respiratory distress, and was hyperactive throughout the exam. She showed brachycephaly, flat occiput, low set ears and a unique structure of the upper eyelids resembling bilateral ptosis. Persistent flexion of the neck raised suspicion for an anomaly of the cervical spine. She further had sparse hair, also notable for a reddish tinge in the hair ends. Skin was translucent with no hypo- or hyperpigmented skin lesions. She had pectus excavatum, and her limbs were notable for brachydactyly in both hands with short fifth digits, syndactyly of II-III toes bilaterally, with metatarsal shortening of the fifth toes. Neurological examination showed no nystagmus, symmetrical facial movements, normal sensation, decreased muscle tone and brisk deep tendon reflexes (DTR) in the lower limbs. Of note, she had an abnormal and unstable walk, although not purely atactic. Her height was 106 cm (3rd centile, Z score -1.83) and head circumference was 45 cm (<1st centile, -4.7 SD).

Previous clinical investigation included a brain CT in early childhood that was reportedly notable for microcephaly and craniosynostosis, and a brain MRI performed at 3 years of age, and considered to demonstrate mild diffuse cortical atrophy. Due to chronic diarrhea and persistent hypereosinophilia and elevated serum IgE levels initially attributed to a *Strongyloides* infection, the patient required several hospitalizations and extensive investigations which were noncontributory. These included abdominal ultrasound, and laboratory tests for immunological and infectious etiologies. Of note, the diarrhea responded to steroid treatment, but repeatedly resumed upon its cessation. Echocardiogram was performed and considered to be normal. She did not have a history of recurrent infections.

Family history is notable for an additional affected elder sister (designated **Patient 5**), 24 years old, who is reportedly similarly but more severely affected. She had developmental delay, and had begun walking at 4 years of age. She shows intellectual disability, facial features resembling those of the proband, and instability when walking, with kyphosis of the cervical spine. She reportedly has spastic paraplegia, and requires a wheelchair when leaving the house.

The other five siblings (males and females) are reportedly healthy, apart from eczema/atopic dermatitis and arthralgia in one male sibling.

Alteration of D-serine and glycine effects on a constitutively open GluD1 ion channel by the R^{161}H and T^{752}M mutations

Our molecular modeling study predicts that R^{161}H and T^{752}M mutations can weaken the binding of endogenous ligands D-serine and glycine to GluD1 (see main text). In order to test this prediction, we expressed in *Xenopus* oocytes the constitutively open channel GluD1^{F655A} mutant (Yadav *et al.*, 2011),

the GluD1$^{F655A+R161H}$ and GluD1$^{F655A+T752M}$ double mutants, as well as a GluD1^{F655A} variant carrying additional mutations that disrupt the D-serine/glycine binding site (Δser-GluD1^{F655A}, see **Suppl. Methods** and Hansen *et al.*, 2009). Spontaneous ion current, recorded upon replacement of the channel-impermeant ion NMDG by Na^+ in the perfusion solution, was observed in oocytes expressing GluD1^{F655A} (638 ± 37 nA, n=38), GluD1$^{F655A+R161H}$ (519 ± 48 nA, n=27), GluD1$^{F655A+T752M}$ (454 ± 54 nA, n=24) and Δser-GluD1^{F655A} (612 ± 58 nA, n=26). The spontaneous current was minimal in oocytes expressing GluD1^{WT} (46 ± 4 nA, n=5), indicating that the current was essentially due to ion permeation through constitutively open channels of GluD1 mutants (**Figure S3**). This was confirmed by application of pentamidine (100 µM), a blocker of GluD channels (Yadav *et al.*, 2011), which reduced spontaneous currents by 38 ± 2 % (GluD1^{F655A}), 34 ± 2 % (GluD1$^{F655A+R161H}$), 32 ± 2 % (GluD1$^{F655A+T752M}$) and 30 ± 2 % (Δser-GluD1^{F655A}). Application of D-Serine (3 mM) and glycine (3 mM) significantly enhanced the spontaneous current in GluD1^{F655A}-expressing oocytes by 8.1 ± 0.4 and 6.1 ± 0.6 %, respectively, but had no measurable effect on Δser-GluD1^{F655A} (**Figure S3**), attesting to their GluD1-specific action. As compared to GluD1^{F655A}, D-serine and glycine enhancement of spontaneous current were little affected for GluD1$^{F655A+R161H}$ (8.8 ± 0.4 % and 5.3 ± 0.5 %, respectively), but were significantly decreased for GluD1$^{F655A+T752M}$ (6.1 ± 0.4 % and 3.9 ± 0.4 %, respectively). These experimental results are consistent with those of the modeling study, and indicate that the T^{752}M mutation can affect GluD1 function by altering ligand binding and its transduction to transmembrane/intracellular signaling.

The R^{161}H and T^{752}M mutations do not hamper cerebellin binding to the extracellular amino-terminal domain of GluD1

The kinetics and affinity of cerebellin binding to GluD1 were investigated using Bio-Layer Interferometry (BLI) measurements on recombinant full-length Cerebellin-1 (Cbln1$_{FL}$) and a fusion of WT or mutant GluD1 amino-terminal and ligand-binding domains with the fragment crystallizable (Fc) region of human IgG1 (WT, R^{161}H or T^{752}M GluD1$_{ATD-LBD}$-Fc, see **Suppl. Methods**). Measurements were performed at seven different Cbln1$_{FL}$ concentrations ranging from 75 to 5000 nM. Consistent with the multimeric nature of cerebellin (Elegheert *et al.*, 2016), association and dissociation kinetics (Ka and Kdis, respectively) were biphasic, and were fitted with a model (see **Suppl. Methods**) yielding two dissociation constants (K_{D1} and K_{D2}) for each Cbln1$_{FL}$ concentration (**Figure S6**). The values of these dissociation constants did not reveal differences in binding affinity between WT, R^{161}H and T^{752}M GluD1$_{ATD-LBD}$-Fc (**Figure S6**) that may hamper cerebellin binding to GluD1 mutants.

Pathophysiological impact of the GluD1^{R161H} and GluD1^{T752M} mutants on dendritic spines

GluD1 is present at excitatory synaptic sites (Konno *et al.*, 2014; Hepp *et al.*, 2015; Benamer *et al.*, 2018), and is able to promote the formation of dendritic spines and excitatory synapses (Ryu *et al.*, 2012; Gupta *et al.*, 2015; Tao *et al.*, 2018, Andrews and Dravid, 2021).

We thus examined the impact of the GluD1 R^{161}H mutation on dendritic spine density and morphology in mature hippocampal primary neuronal cultures from *Grid1*$^{+/+}$ mice using co-transfection of plasmids encoding HA-GluD1^{WT} or HA-GluD1^{R161H} together with a GFP-expressing plasmid. We found a significant increase in the density of dendritic spines in neurons overexpressing GluD1^{WT} as compared to GFP-only, control neurons (spine number per 10 µm dendritic segment; control: 5.3 ± 0.2, n=6 neurons; GluD1^{WT}: 7.0 ± 0.3, n=9 neurons), consistent with the reported spine-promoting function of GluD1 (Gupta *et al.*, 2015). Conversely, neurons overexpressing GluD1^{R161H} exhibited a spine density (5.2 ± 0.2 per 10 µm segment, n=8 neurons) similar to that of control neurons (**Figure S8**). We also observed, in the same dendritic sections, that the proportion of immature spines (see **Suppl. Methods**

and Zagrebelsky *et al.*, 2005) was significantly enhanced in $GluD1^{R161H}$-transfected neurons as compared to control neurons (control: 20.1 ± 2.3, $GluD1^{WT}$: 23.4 ± 1.6, $GluD1^{R161H}$: 28.3 ± 1.4 %; $GluD1^{R161H}$>control, $p<0.05$; **Figure S8**). These results indicate that the $R^{161}H$ mutation impairs GluD1 stimulatory effects on dendritic spine formation and maturation.

The impact of the GluD1 $R^{161}H$ and $T^{752}M$ mutations on dendritic spine density was also investigated in mouse hippocampal organotypic slice cultures using single cell co-electroporation of plasmids encoding $GluD1^{WT}$, $GluD1^{R161H}$ or $GluD1^{T752M}$ together with a tdTomato-expressing plasmid (see **Suppl. Methods**). CA1 hippocampal neurons overexpressing $GluD1^{WT}$ exhibited significantly higher spine density (tdTomato+$GluD1^{WT}$: 1.02 ± 0.04 per µm, n=48 segments of apical dendrites) than tdTomato-only (tdTomato: 0.71 ± 0.04 per µm, n=20), $GluD1^{R161H}$–overexpressing (tdTomato+$GluD1^{R161H}$: 0.83 ± 0.03 per µm, n=63) and $GluD1^{T752M}$–overexpressing (tdTomato+$GluD1^{T752M}$: 0.71 ± 0.04 per µm, n=45) neurons (**Figure S11**). Moreover, the spine density of neurons overexpressing $GluD1^{R161H}$ or $GluD1^{T752M}$ did not significantly differ from that of tdTomato-only neurons ($p=0.11$ and $p>0.99$, respectively). These results confirm, in a more integrated neural environment, that the $R^{161}H$ and $T^{752}M$ mutations impair GluD1 stimulatory effects on dendritic spine formation.

Supplementary References

Andrews PC, Dravid SM. An emerging map of glutamate delta 1 receptors in the forebrain. Neuropharmacology. 2021 Jul 1;192:108587.

Behiels E, Elegheert J. High-Level Production of Recombinant Eukaryotic Proteins from Mammalian Cells Using Lentivirus. Methods Mol Biol. 2021;2305:83-104.

Benamer N, Marti F, Lujan R, Hepp R, Aubier TG, Dupin AAM, Frébourg G, Pons S, Maskos U, Faure P, Hay YA, Lambolez B, Tricoire L. GluD1, linked to schizophrenia, controls the burst firing of dopamine neurons. Mol Psychiatry. 2018 Mar;23(3):691-700.

Bonnot A, Guiot E, Hepp R, Cavellini L, Tricoire L, Lambolez B. Single-fluorophore biosensors based on conformation-sensitive GFP variants. FASEB J. 2014 Mar;28(3):1375-85.

Burada AP, Vinnakota R, Kumar J. Cryo-EM structures of the ionotropic glutamate receptor GluD1 reveal a non-swapped architecture. Nat Struct Mol Biol. 2020 Jan;27(1):84-91.

Dhers L, Pietrancosta N, Ducassou L, Ramassamy B, Dairou J, Jaouen M, André F, Mansuy D, Boucher JL. Spectral and 3D model studies of the interaction of orphan human cytochrome P450 2U1 with substrates and ligands. Biochim Biophys Acta Gen Subj. 2017 Jan;1861(1 Pt A):3144-3153.

Drobac E, Tricoire L, Chaffotte AF, Guiot E, Lambolez B. Calcium imaging in single neurons from brain slices using bioluminescent reporters. J Neurosci Res. 2010 Mar;88(4):695-711.

Ducassou L, Jonasson G, Dhers L, Pietrancosta N, Ramassamy B, Xu-Li Y, Loriot MA, Beaune P, Bertho G, Lombard M, Mansuy D, André F, Boucher JL. Expression in yeast, new substrates, and construction of a first 3D model of human orphan cytochrome P450 2U1: Interpretation of substrate hydroxylation regioselectivity from docking studies. Biochim Biophys Acta. 2015 Jul;1850(7):1426-37.

Elegheert J, Kakegawa W, Clay JE, Shanks NF, Behiels E, Matsuda K, Kohda K, Miura E, Rossmann M, Mitakidis N, Motohashi J, Chang VT, Siebold C, Greger IH, Nakagawa T, Yuzaki M, Aricescu AR. Structural basis for integration of GluD receptors within synaptic organizer complexes. Science. 2016 Jul 15;353(6296):295-9.

Elegheert J, Behiels E, Bishop B, Scott S, Woolley RE, Griffiths SC, Byrne EFX, Chang VT, Stuart DI, Jones EY, Siebold C, Aricescu AR. Lentiviral transduction of mammalian cells for fast, scalable and high-level production of soluble and membrane proteins. Nat Protoc. 2018 Dec;13(12):2991-3017.

Ferreira TA, Blackman AV, Oyrer J, Jayabal S, Chung AJ, Watt AJ, Sjöström PJ, van Meyel DJ. Neuronal morphometry directly from bitmap images. Nat Methods. 2014 Oct;11(10):982-4.

Gao J, Maison SF, Wu X, Hirose K, Jones SM, Bayazitov I, Tian Y, Mittleman G, Matthews DB, Zakharenko SS, Liberman MC, Zuo J. Orphan glutamate receptor delta1 subunit required for high-frequency hearing. Mol Cell Biol. 2007 Jun;27(12):4500-12.

García-Nafría J, Watson JF, Greger IH. IVA cloning: A single-tube universal cloning system exploiting bacterial In Vivo Assembly. Sci Rep. 2016 Jun 6;6:27459.

Gervasi N, Hepp R, Tricoire L, Zhang J, Lambolez B, Paupardin-Tritsch D, Vincent P. Dynamics of protein kinase A signaling at the membrane, in the cytosol, and in the nucleus of neurons in mouse brain slices. J Neurosci. 2007 Mar 14;27(11):2744-50.

Gupta SC, Yadav R, Pavuluri R, Morley BJ, Stairs DJ, Dravid SM. Essential role of GluD1 in dendritic spine development and GluN2B to GluN2A NMDAR subunit switch in the cortex and hippocampus reveals ability of GluN2B inhibition in correcting hyperconnectivity. Neuropharmacology 2015; 93: 274-84.

Hansen KB, Naur P, Kurtkaya NL, Kristensen AS, Gajhede M, Kastrup JS, Traynelis SF. Modulation of the dimer interface at ionotropic glutamate-like receptor delta2 by D-serine and extracellular calcium. J Neurosci. 2009 Jan 28;29(4):907-17.

Hepp R, Hay YA, Aguado C, Lujan R, Dauphinot L, Potier MC, et al. Glutamate receptors of the delta family are widely expressed in the adult brain. Brain Struct Funct 2015; 220: 2797-815.

Jo, S.; Kim, T.; Iyer, V. G.; Im, W. CHARMM-GUI: A Web-Based Graphical User Interface for CHARMM. J. Comput. Chem. 2008, 29, 1859– 1865, DOI: 10.1002/jcc.20945

Konno K, Matsuda K, Nakamoto C, Uchigashima M, Miyazaki T, Yamasaki M, et al. Enriched expression of GluD1 in higher brain regions and its involvement in parallel fiber-interneuron synapse formation in the cerebellum. J Neurosci 2014; 34: 7412-24.

Perroy J, Raynaud F, Homburger V, Rousset MC, Telley L, Bockaert J et al. Direct interaction enables cross-talk between ionotropic and group I metabotropic glutamate receptors. J Biol Chem 2008; 283(11): 6799-6805.

Pologruto TA, Sabatini BL, Svoboda K. ScanImage: flexible software for operating laser scanning microscopes. Biomed Eng Online. 2003 May 17;2:13.

Ryu K, Yokoyama M, Yamashita M, Hirano T. Induction of excitatory and inhibitory presynaptic differentiation by GluD1. Biochem Biophys Res Commun 2012; 417: 157-61.

Schindelin J, Arganda-Carreras I, Frise E, Kaynig V, Longair M, Pietzsch T, Preibisch S, Rueden C, Saalfeld S, Schmid B, Tinevez JY, White DJ, Hartenstein V, Eliceiri K, Tomancak P, Cardona A. Fiji: an open-source platform for biological-image analysis. Nat Methods. 2012 Jun 28;9(7):676-82.

Schneider CA, Rasband WS, Eliceiri KW. NIH Image to ImageJ: 25 years of image analysis. Nat Methods. 2012 Jul;9(7):671-5.

Stoppini L, Buchs PA, Muller D. A simple method for organotypic cultures of nervous tissue. J Neurosci Methods. 1991 Apr;37(2):173-82.

Tao W, Díaz-Alonso J, Sheng N, Nicoll RA. Postsynaptic δ1 glutamate receptor assembles and maintains hippocampal synapses via Cbln2 and neurexin. Proc Natl Acad Sci U S A. 2018 Jun 5;115(23):E5373-E5381.

Thestrup T, Litzlbauer J, Bartholomäus I, Mues M, Russo L, Dana H, Kovalchuk Y, Liang Y, Kalamakis G, Laukat Y, Becker S, Witte G, Geiger A, Allen T, Rome LC, Chen TW, Kim DS, Garaschuk O, Griesinger C, Griesbeck O. Optimized ratiometric calcium sensors for functional in vivo imaging of neurons and T lymphocytes. Nat Methods. 2014 Feb;11(2):175-82.

Umesono K, Murakami KK, Thompson CC, Evans RM. Direct repeats as selective response elements for the thyroid hormone, retinoic acid, and vitamin D3 receptors. Cell. 1991 Jun 28;65(7):1255-66.

Ung DC, Iacono G, Méziane H, Blanchard E, Papon MA, Selten M, van Rhijn JR, Montjean R, Rucci J, Martin S, Fleet A, Birling MC, Marouillat S, Roepman R, Selloum M, Lux A, Thépault RA, Hamel P, Mittal K, Vincent JB, Dorseuil O, Stunnenberg HG, Billuart P, Nadif Kasri N, Hérault Y, Laumonnier F. Ptchd1 deficiency induces excitatory synaptic and cognitive dysfunctions in mouse. Mol Psychiatry. 2018 May;23(5):1356-1367.

Villmann C, Strutz N, Morth T, Hollmann M. Investigation by ion channel domain transplantation of rat glutamate receptor subunits, orphan receptors and a putative NMDA receptor subunit. Eur J Neurosci. 1999 May;11(5):1765-78. doi: 10.1046/j.1460-9568.1999.00594.x. PMID: 10215929.

Wu G, Robertson DH, Brooks CL 3rd, Vieth M. Detailed analysis of grid-based molecular docking: A case study of CDOCKER-A CHARMm-based MD docking algorithm. J Comput Chem. 2003 Oct;24(13):1549-62. doi: 10.1002/jcc.10306. PMID: 12925999.

Yadav R, Rimerman R, Scofield MA, Dravid SM. Mutations in the transmembrane domain M3 generate spontaneously open orphan glutamate δ1 receptor. Brain Res. 2011 Mar 25;1382:1-8.

Zagrebelsky M, Holz A, Dechant G, Barde YA, Bonhoeffer T, Korte M. The p75 neurotrophin receptor negatively modulates dendrite complexity and spine density in hippocampal neurons. J Neurosci. 2005 Oct 26;25(43):9989-99.

Supplementary Figure Legends

Supplementary Figure 1: Genome-wide homozygosity mapping data in family A

The Y axis represents the LOD score and the X axis represents the genetic distance (chromosomes). Two regions have a maximum LOD score, the largest within chromosome 10 (encompassing *GRID1*) and the second one within the telomeric region of the long arm of chromosome 12.

Supplementary Figure 2: Arrangement of extracellular domains in the GluD1 homotetramer

Modelled complete structure of the GluD1 homotetramer derived from Burada *et al.* (2020) and including the newly generated 3D loops (yellow) between ATD and LBD, and between LBD and transmembrane domains.

Supplementary Figure 3: Alteration of D-serine and glycine effects on a constitutively open GluD1 ion channel by the R161H and T752M mutations

(A) Examples of spontaneous Na^+ currents recorded in *Xenopus* oocytes expressing indicated GluD1 isoforms and effects of D-serine (3 mM), glycine (3 mM) and pentamidine (100 µM). **(B)** Mean amplitudes of spontaneous Na^+ currents and of their inhibition by pentamidine. **(C)** Mean amplitudes of D-serine- and glycine-induced currents normalized to spontaneous Na^+ currents. *Significant, n.s. differences

Supplementary Figure 4: The $R^{161}H$ and $T^{752}M$ mutations do not hamper the expression and trafficking to the plasma membrane of GluD1

(A) Immunoblots of protein lysates (total, cytosolic and membranes fractions) extracted from HEK cells expressing HA-$GluD1^{WT}$, HA-$GluD1^{R161H}$, or HA-$GluD1^{T752M}$ (predicted molecular weight 110 kDa). Beta-actin was used as protein loading control. Similar results were obtained in n=3 independent experiments in each condition. **(B)** Confocal microscopy images of spiny hippocampal neurons from primary cell cultures transfected with plasmids encoding HA-$GluD1^{WT}$, HA-$GluD1^{R161H}$, or HA-$GluD1^{T752M}$, and revealed using anti-HA immunostaining. A zoomed area of a dendritic section is presented for each condition. Similar results were obtained on at least 9 neurons examined from n=3 independent experiments in each condition.

Supplementary Figure 5: The $R^{161}H$ and $T^{752}M$ mutations do not hinder cerebellin binding to GluD1

Fluorescence pictures of HEK cells expressing $GluD1^{WT}$, $GluD1^{R161H}$, or $GluD1^{T752M}$ and incubated with HA-tagged cerebellin (HA-Cbln1) prior to fixation, immunolabelling of GluD1 and HA-Cbln1, and nuclear staining with DAPI. Note that HA-Cbln1 immunostaining was similar for $GluD1^{WT}$-, $GluD1^{R161H}$-, and $GluD1^{T752M}$-expressing cells, and that HA-Cbln1 binding was not detected on $GluD1^{WT}$-, $GluD1^{R161H}$-, and $GluD1^{T752M}$-negative cells. Similar results were obtained in n=3 independent experiments in each condition.

Supplementary Figure 6: Direct interaction analysis of $Cbln1_{FL}$ with $GluD1_{ATD-LBD}$ WT, $R^{161}H$ and $T^{752}M$

(A) Schematic representation of the Bio-Layer Interferometry (BLI) experimental setup. Fc-linked $GluD1_{ATD-LBD}$ WT, R161H and T752M proteins were coupled to the sensor surfaces, and subsequently exposed to a two-fold dilution series of $Cbln1_{FL}$. **(B)** Individual BLI sensorgrams are color-coded according to $Cbln1_{FL}$ concentration, and kinetic fits (obtained using a 2:1 heterogeneous binding model to account for avidity effects) are shown as black lines. K_{D1} and K_{D2} values for each concentration are

annotated. For each dataset, the fitted K_{a1}/K_{dis1} and K_{a2}/K_{dis2} association/dissociation values for the individual concentrations are plotted onto iso-affinity graphs.

Supplementary Figure 7: The GluD1 R^{161}H and T^{752}M mutations do not hamper mGlu1-GluD1 physical interaction

(A) Fluorescence pictures of HEK cells co-expressing GluD1^{WT}, GluD1^{R161H}, or GluD1^{T752M} and HA-tagged mGlu1. **(B)** *Left panel*: HEK cell lysates were subjected to immunoprecipitation (IP) with indicated antibodies against GluD1, HA-tagged mGlu1, or with a control antibody (IgG). Immunoblotting (IB) of IP eluates or cell lysates were probed using indicated antibodies. *Right panel*: The bar graph summarizes results of 3 experiments performed in duplicate for each mGlu1+GluD1^{WT}/GluD1^{R161H}/GluD1^{T752M} combination. The mean intensity of the bands GluD1^{WT} pulled down by mGlu1 and of the bands mGlu1 pulled down by GluD1^{WT} was normalized to 100%. Results of experiments involving GluD1 mutants are expressed as % of results involving GluD1^{WT}.

Supplementary Figure 8: Lentiviral transfer of GluD1-ires-GFP in neurons of primary cortical cell cultures from *Grid1*$^{-/-}$ mice

Fluorescence pictures of a primary cortical cell culture transduced with a lentivirus co-expressing GluD1 and GFP, and processed for immunolabelling of GFP and the neuronal marker MAP2, and for nuclear staining with DAPI. The graph summarizes results obtained on 6823 DAPI-positive cells from 2 cultures, 8 coverslips, 5 area analyzed per coverslip. Among DAPI-positive cells, 35 ± 1 % were GFP-positive, and 28 ± 1 % were MAP2-positive. Note that 71 ± 3 % of GFP-positive cells were Map2-positive, and that 88 ± 2 % of MAP2-positive cells were GFP-positive, showing that GluD1-expressing lentiviruses preferentially and efficiently transduced neurons.

Supplementary Figure 9: Expression of transfected GluD1^{WT} and GluD1 mutants is largely superior to endogenous GluD1 in *Grid1*$^{+/+}$ hippocampal neurons

Fluorescence pictures showing hippocampal neurons in culture immunostained for GFP, GluD1 and the neuronal marker MAP2 after transfection of indicated plasmids. Note that transfected cells express MAP2, and that expression of transfected GluD1 is far superior to endogenous GluD1, as evidenced by the very faint immunostaining of non-transfected neurons.

Supplementary Figure 10: Pathophysiological impact of the GluD1^{R161H} mutant on dendritic spines of dissociated hippocampal neurons in primary cultures.

(A) *Upper panels*: Confocal microscopy images of cultured hippocampal neurons expressing GFP alone, or both GFP and HA-GluD1^{WT} or HA-GluD1^{R161H}, after plasmid transfection. All GFP-expressing neurons examined after co-transfection also expressed either HA-GluD1^{WT} (n=64) or HA-GluD1^{R161H}, (n=64) as shown by anti-HA immunostaining of 5 independent culture transfections. *Lower panels*: Confocal microscopy images of spiny dendritic sections of hippocampal neurons transfected as indicated. **(B)** Graphs summarizing results of spine density and morphology analyses performed on n=6 (GFP), 9 (GFP+HA-GluD1^{WT}), and 8 (GFP+HA-GluD1^{R161H}) neurons from at least 3 independent cultures in each condition, and using mean values obtained from at least 2 dendritic segments per neuron. Immature spines comprise thin-long and filopodia-shaped spines, as opposed to mushroom-shaped and stubby mature spines. *Significant differences

Supplementary Figure 11: Pathophysiological impact of GluD1^{R161H} and GluD1^{T752M} mutants on the synaptic density of hippocampal CA1 pyramidal neurons in organotypic slice culture

(A) Confocal images of CA1 pyramidal neurons from hippocampal organotypic slices electroporated with tdTomato (magenta) and HA-GluD1^{WT}and immunostained with anti-HA antibody (green). **(B)**

Confocal images of apical dendrites from CA1 pyramidal neurons electroporated with tdTomato (magenta) alone or together with $GluD1^{WT}$, $GluD1^{R161H}$ or $GluD1^{T752M}$. **(C)** Graph summarizing spine density measured on n=20 (tdTomato), 48 (tdTomato+$GluD1^{WT}$), 63 (tdTomato+$GluD1^{R161H}$) and 45 (tdTomato+ $GluD1^{T752M}$) segments of apical dendrites from at least 3 slices from different mice in each condition. *Significant differences

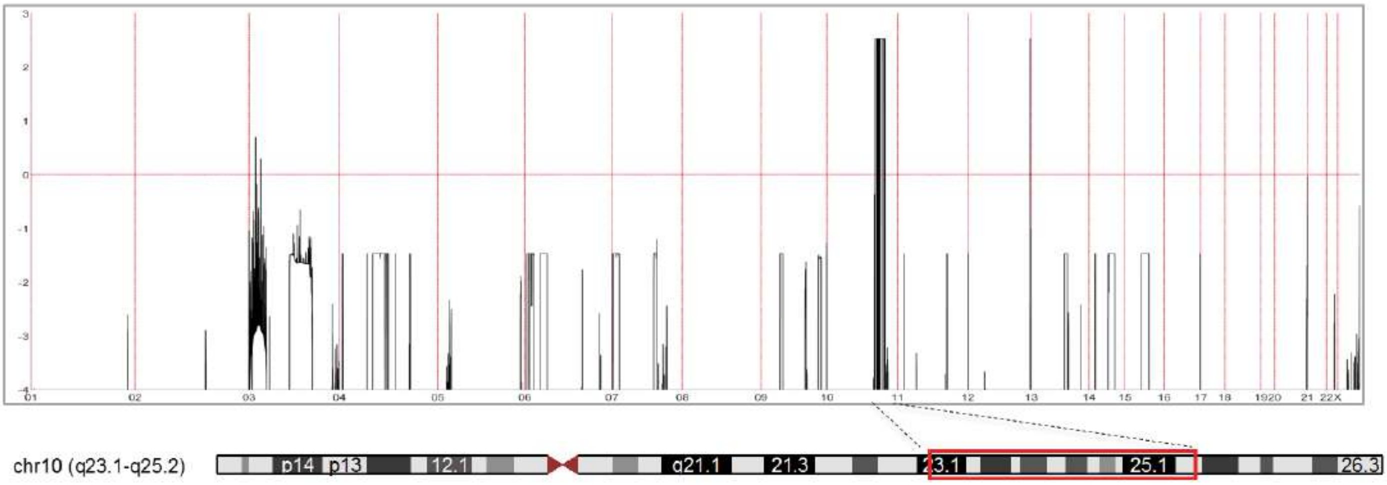

Suppl. Figure 1

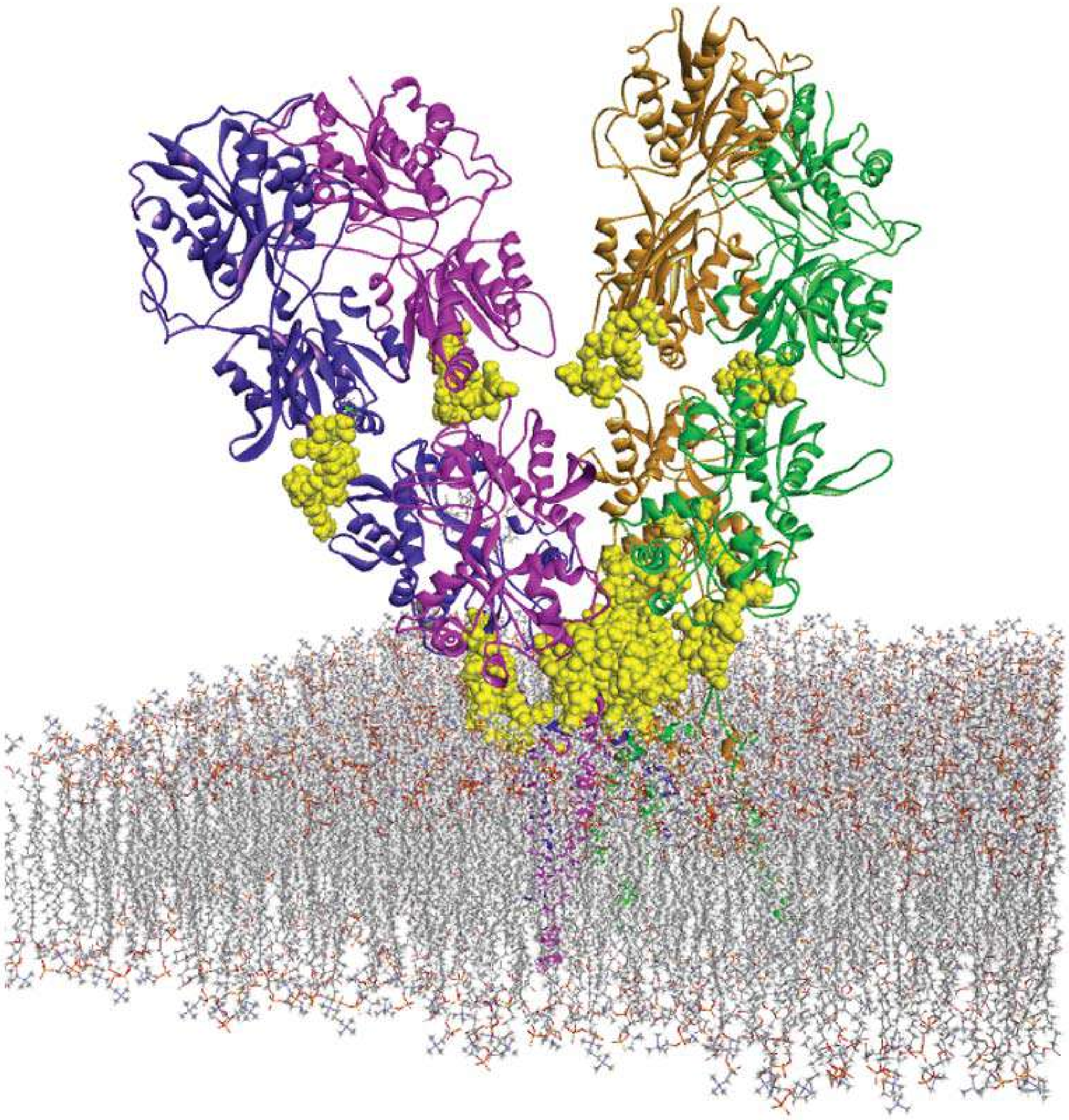

Suppl. Figure 2

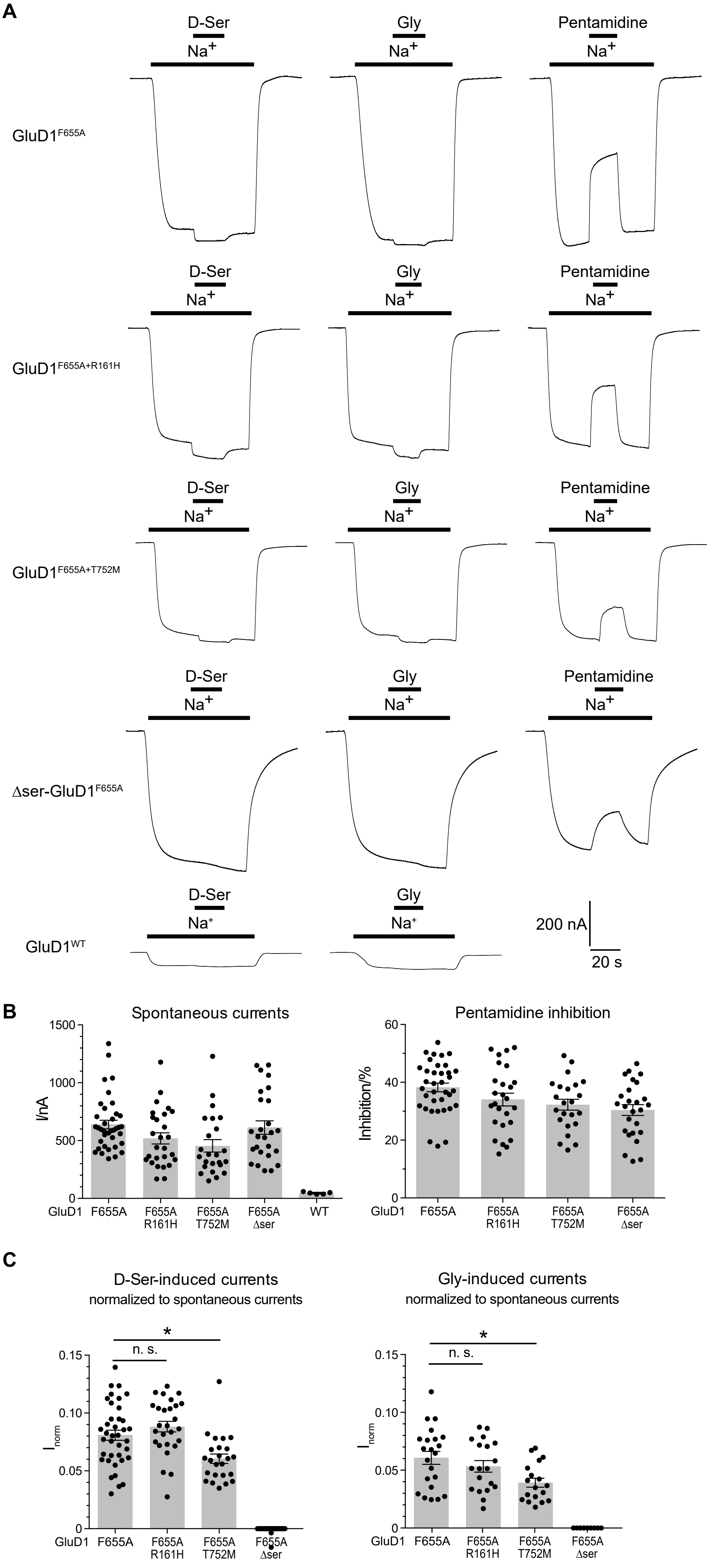

Supp figure 3

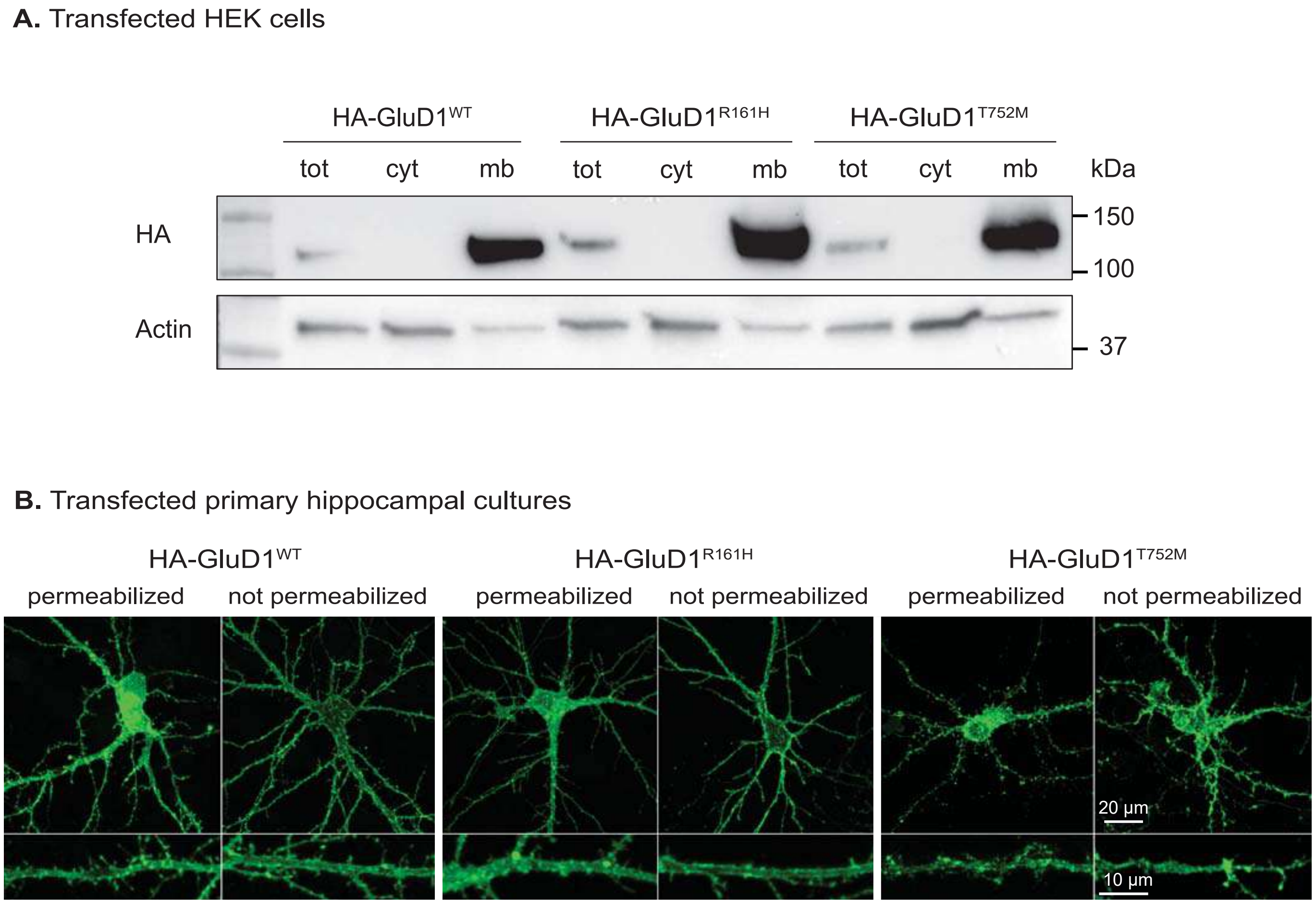

Suppl. Figure 4

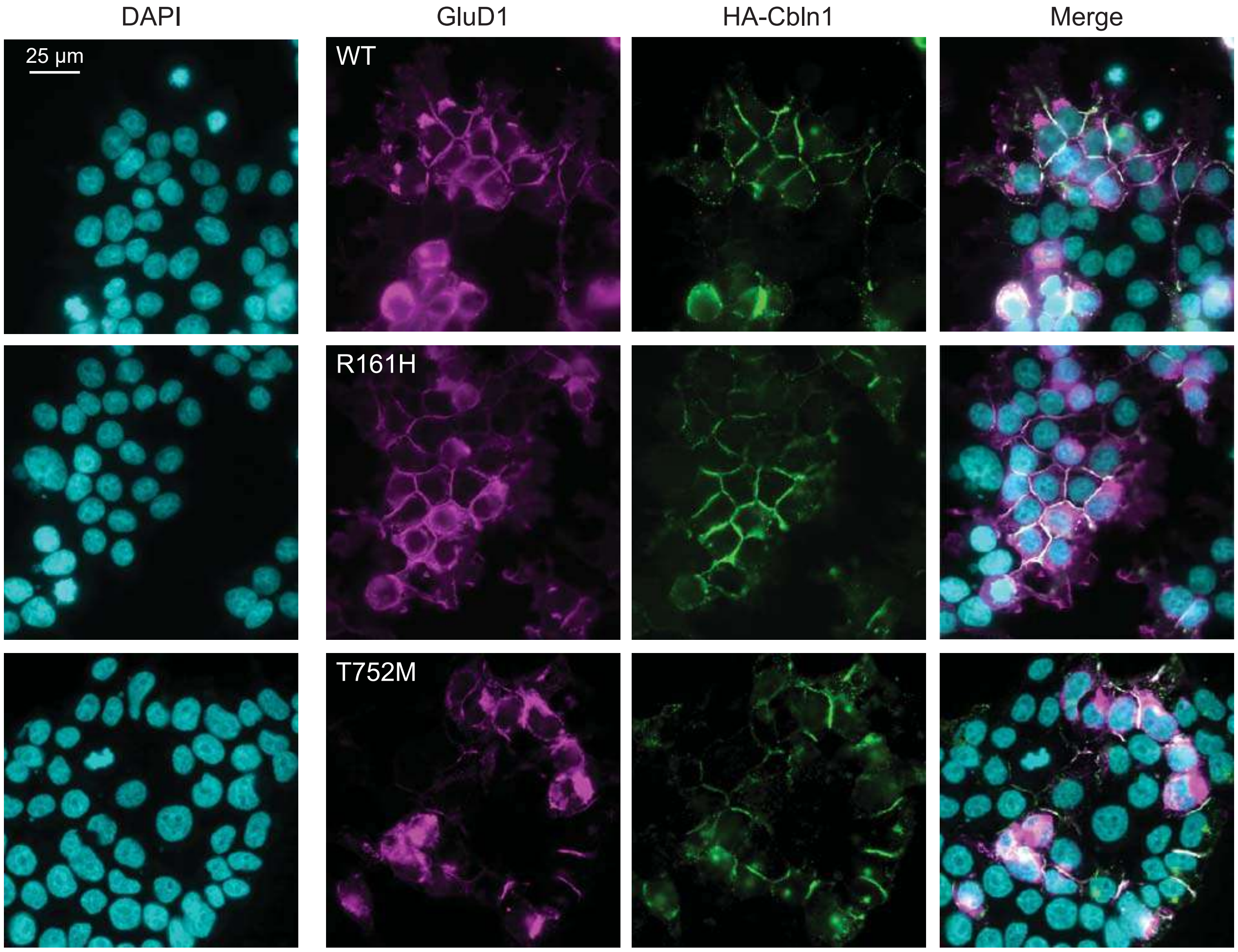

Suppl. Figure 5

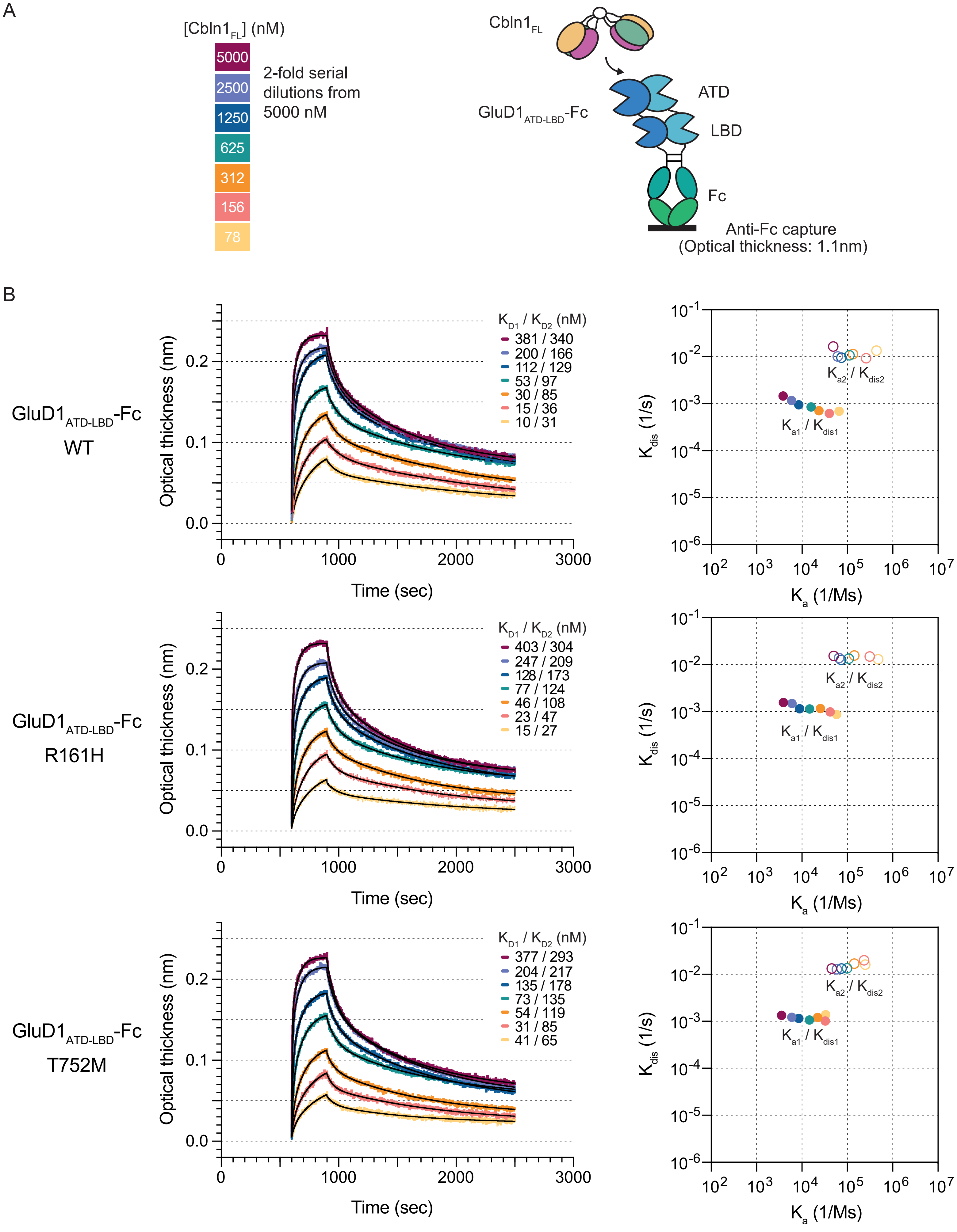

Suppl. figure 6

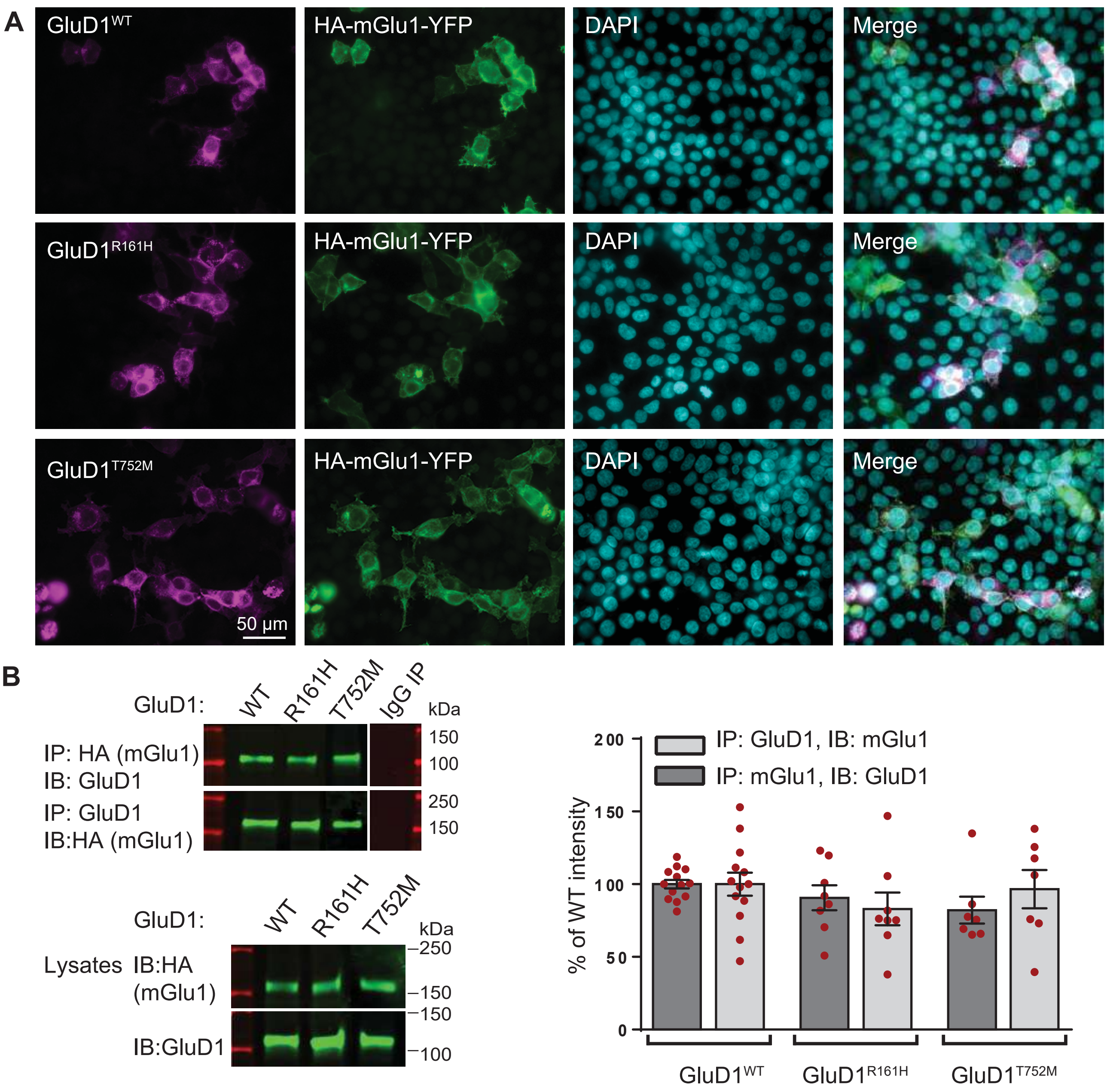

Suppl. Figure 7

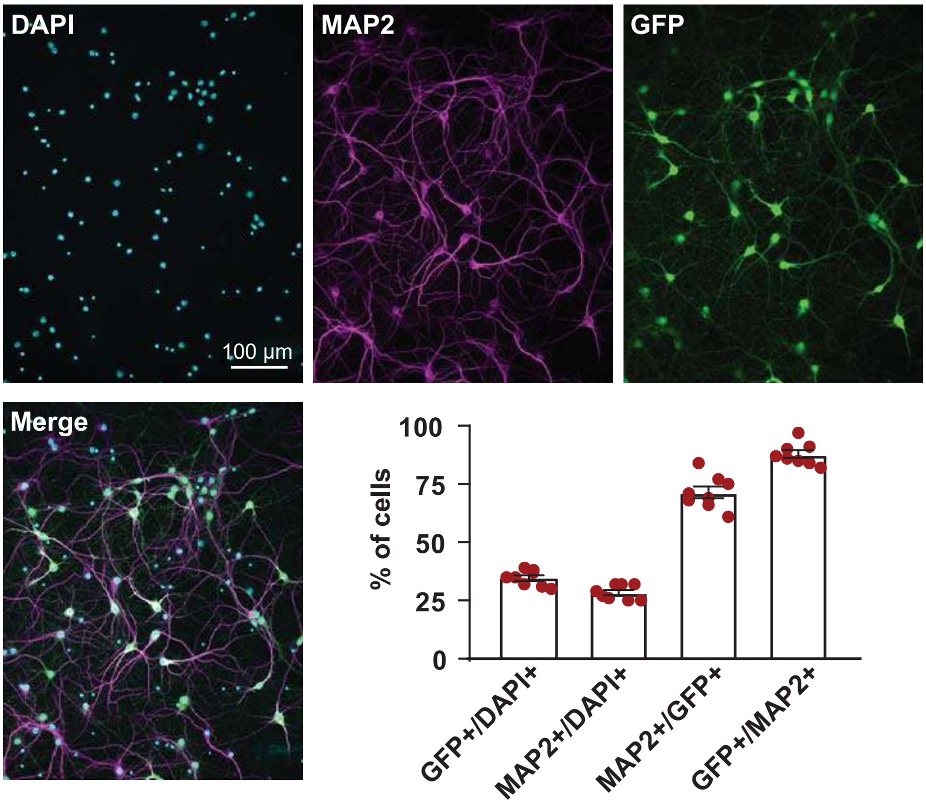

Suppl. Figure 8

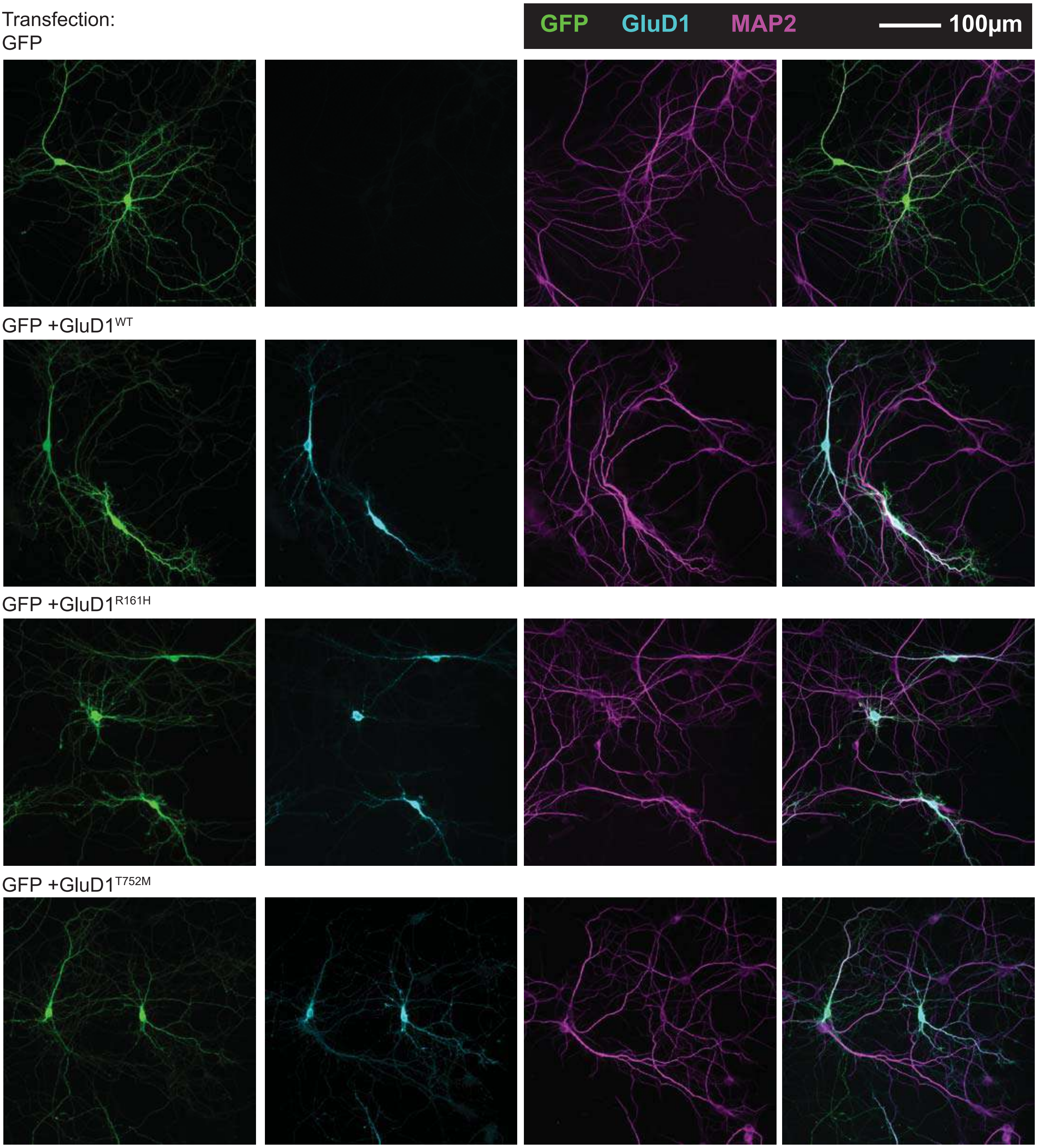

Suppl. Figure 9

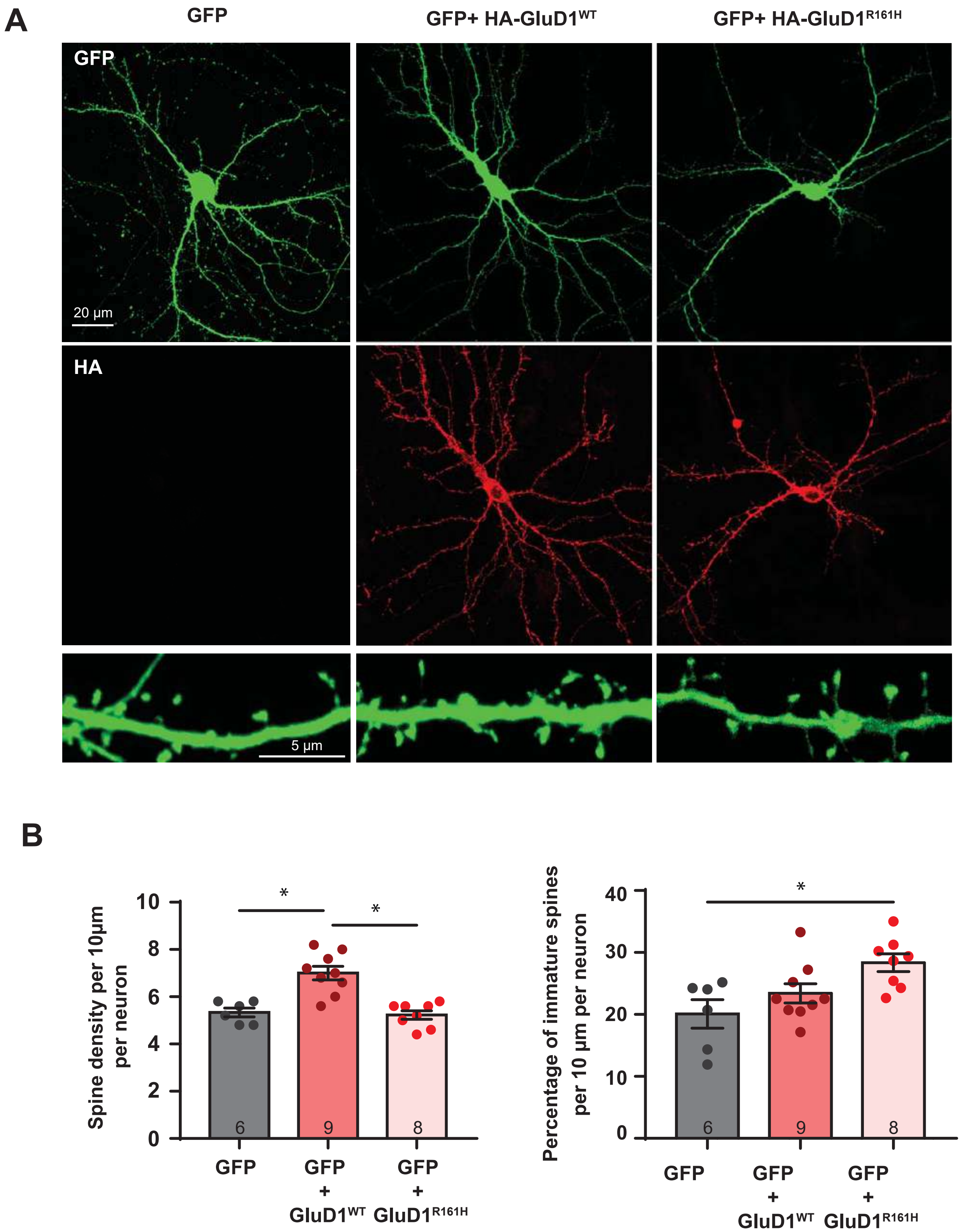

Suppl. Figure 10

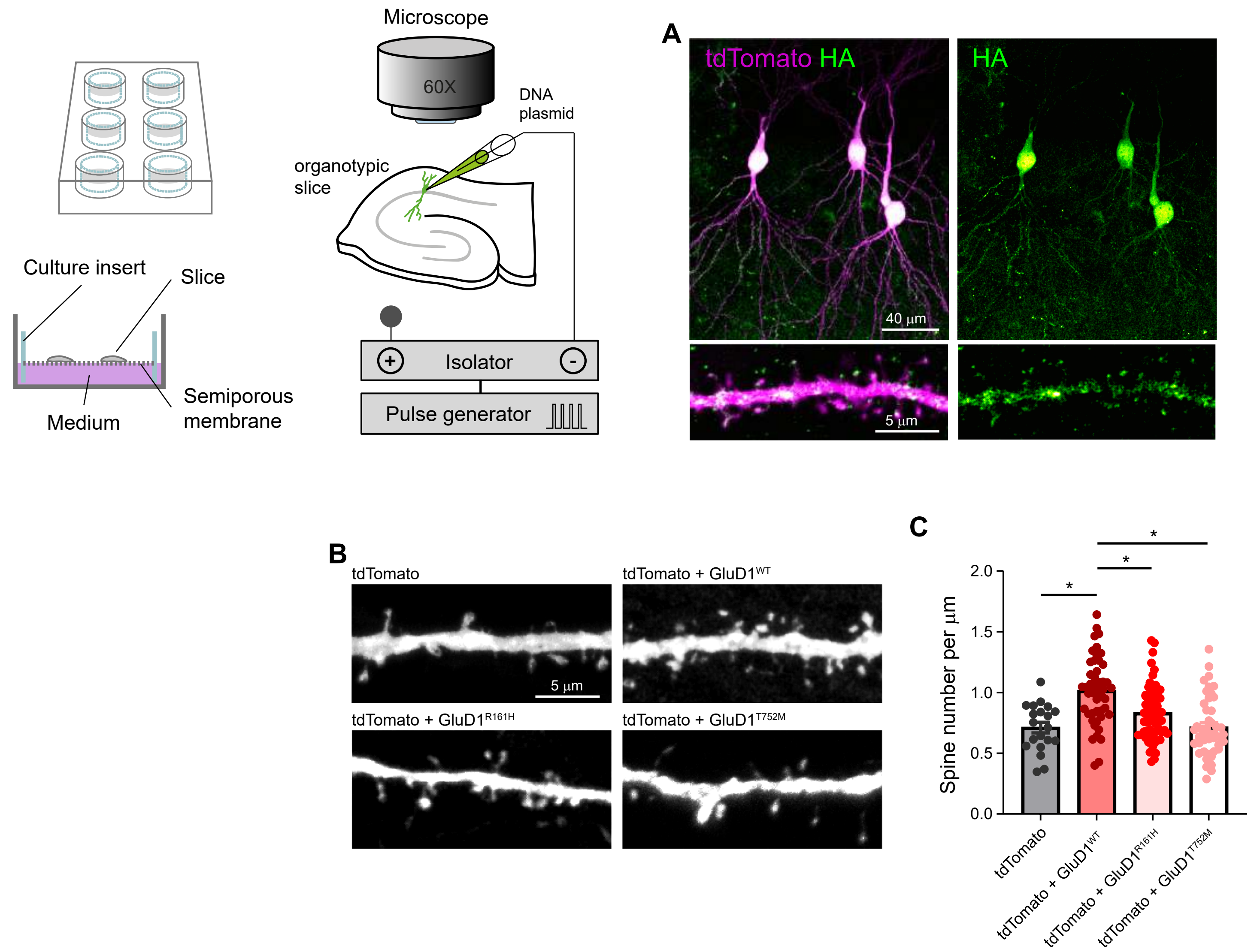

Suppl. Figure 11
